# Fidelity and Promiscuity in an Ant-Plant Mutualism: A Case Study of *Triplaris* and *Pseudomyrmex*

**DOI:** 10.1371/journal.pone.0143535

**Published:** 2015-12-02

**Authors:** Adriana Sanchez

**Affiliations:** Programa de Biología, Universidad del Rosario, Bogotá, Colombia; Smithsonian National Museum of Natural History, UNITED STATES

## Abstract

The association between the myrmecophyte *Triplaris* and ants of the genus *Pseudomyrmex* is an often-reported example of mutualism but no molecular studies have examined this association to date. In this study, the interspecific relationships of *Triplaris* were reconstructed using five molecular markers (two chloroplast and three nuclear), and the relationships of the associated *Pseudomyrmex* using two molecular regions (one mitochondrial and one nuclear). A data set including all known collections of plant hosts and resident ants was also compiled. The pattern of distribution of both organisms reveals that there are varying degrees of host specificity; most ants show broader host usage (promiscuous) but one species (*P*. *dendroicus*) is faithful to a single species of *Triplaris*. In most ant-plant interactions, host usage is not specific at the species level and preferences may result from geographical or ecological sorting. The specificity of *P*. *dendroicus* could be based on chemical recognition of the host they were raised on.

## Introduction

Myrmecophytism, or plants that associate with ants, are a common symbiosis in nature, pervasive and diverse in the tropics, especially in the Neotropics (more than 200 species; [1]). This is a mutualistic association as, in return for being housed and sometimes fed, plant-ants protect their host myrmecophyte from encroaching vegetation, herbivores and pathogens, and/or provide them with nutrients (i.e., myrmecotrophy) [2, 3]. In almost all myrmecophytes, multiple ant queens often colonize different modules of the same plant, which can lead to strong intra- and interspecific competition [4]. Strong competition can potentially result in ecological sorting [5] but other factors such as host-switching, secondary colonization and ecological replacement can modify the associations [4]. Ants may also be more sensitive to habitat than to taxonomic differences among symbiotic partners [6, 7], and/or they could be affected by the demographic and life-history characteristics of the plant and ant populations [8].

There are several well-known cases of these associations, such as the interaction between *Vachellia-Pseudomyrmex*, *Cecropia-Azteca*, and *Macaranga-Crematogaster*. Most studies on these interactions focus on ecological aspects such as defense against herbivores and the effect of different ant genera or species on the hosts (e.g., [9, 10, 11]); however, fewer studies have addressed phylogenetic aspects of the interactions. Phylogenetic studies on ant-plant relationships have shown that there is no support for strict cospeciation between ants and plants (e.g., [12, 13, 14, 15, 16, 17, 18, 19]). In many cases myrmecophytism has evolved multiple times in a single genus (e.g., *Macaranga*; [15]) and in other cases it has evolved in just one clade (i.e., *Vachellia* [19, 20]). However, the system tends to be “promiscuous” since a host species can associate with different obligate ant species, and ant species can colonize different hosts.

The *Triplaris*-*Pseudomyrmex* interaction is a well-known case of myrmecophytism in the Neotropics and some ecological studies have addressed the interaction [21, 22, 23, 24, 25, 26, 27]. However, although there has been some work done on the relationships between the *Pseudomyrmex* species associated with *Triplaris* [28], there has been no attempt to compare the phylogenetic framework of these two groups of organisms.

**Fig 1:**
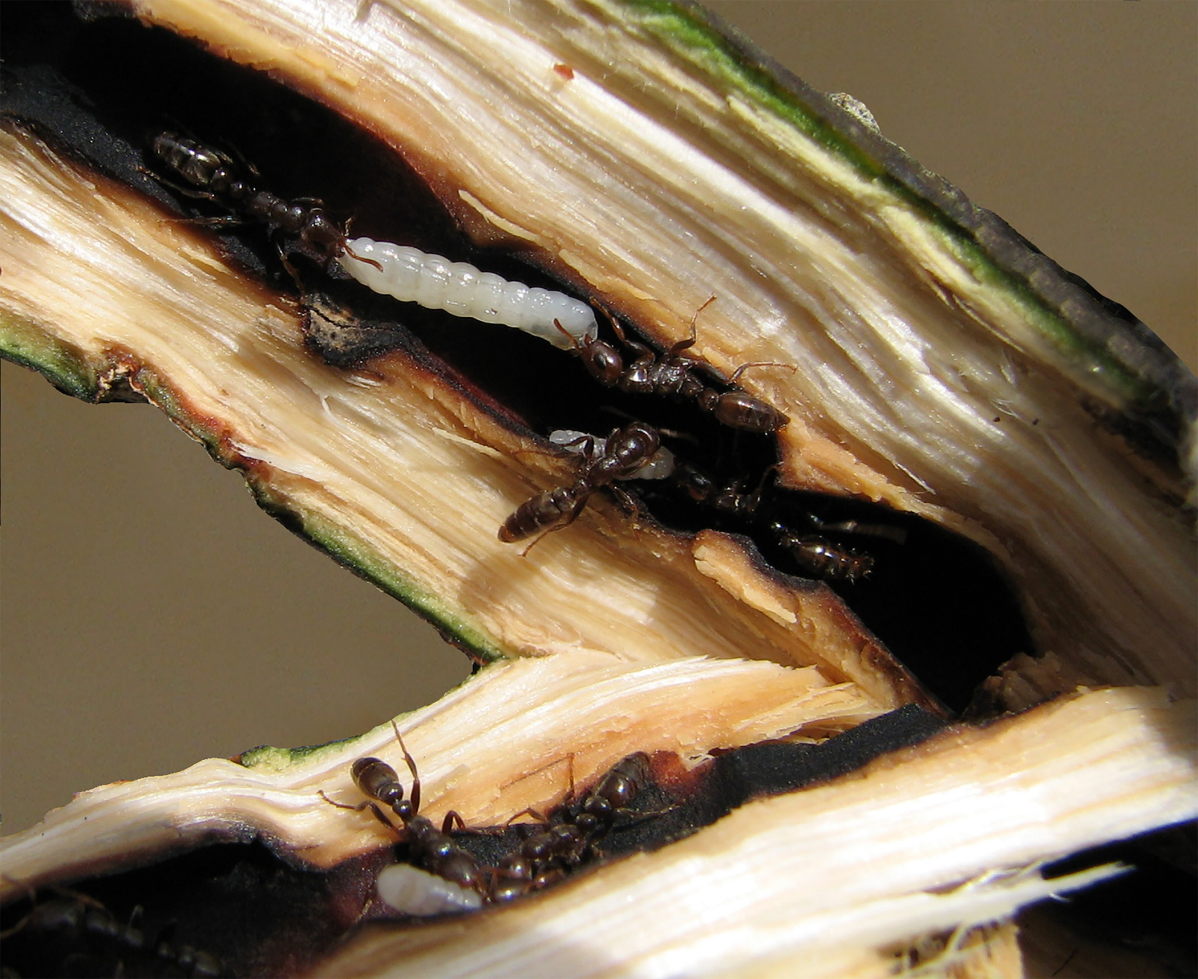
Mutualism between *Triplaris* and *Pseudomyrmex*. Ants of *P*. *triplaridis* establish their colonies in the hollow stems of *T*. *americana*.

*Triplaris* Loefl. (Eriogonoideae, Polygonaceae) is a genus that includes 18 dioecious species of trees with a Neotropical distribution from southern Mexico to southern Brazil. All species occur in lowland habitats from sea level to 2000 m in altitude and are considered important components in all stages of secondary succession [29]. The taxonomy of the genus was revised by Brandbyge [29], where he reduced the 73 taxa described to 18 species (and one subspecies; [29, 30]), but no phylogenetic studies have addressed the interspecific relationships of *Triplaris* to date. A conspicuous feature of all species in *Triplaris* is the hollow stems that harbor ants (Fig 1). Although the plants produce no food bodies or extra-floral nectaries, rewards to the ants are provided by a third symbiont—scale insects (Coccidea, Hemiptera) in the form of honeydew. *Triplaris* is mainly colonized by a group of ant partners belonging to the large and New World-distributed genus *Pseudomyrmex* Lund. (200 species; Pseudomyrmecinae; Fig 1). These ants are characterized by possessing large eyes, aggressive behavior and a painful sting [31]. Six species of *Pseudomyrmex* in the *triplarinus* subgroup have been recognized as obligate and specific mutualists to *Triplaris* (specialized ants that have not been found nesting outside their plants; [28]): *P*. *dendroicus* Forel, *P*. *mordax* Warming, *P*. *triplaridis* Forel, *P*. *triplarinus* Weddell, *P*. *ultrix* Ward, *P*. *vitabilis* Ward. These species are distributed from southern Panama to southern Brazil. Some ecological experiments have studied the symbiosis between *Triplaris* and *Pseudomyrmex*, and it is thought that in exchange for the nesting sites, the ant partners protect their host plants against herbivore damage [23, 27, 32] and maintain the plants free of pathogens. *Pseudomyrmex* is also known to prune the vegetation around their host [23]. Some other ant genera can opportunistically colonize *Triplaris* and it is not uncommon to find non-specialist ant genera such as *Azteca*, *Camponotus*, *Cephalotes*, *Crematogaster*, *Dolichoderus*, and *Pheidole*, as well as less specialized species of *Pseudomyrmex* (e.g., *P*. *elongatus*, *P*. *gebellii*, *P*. *longior*, *P*. *rubiginosus*, and *P*. *viduus*; [24, 28, 33], A. Sanchez personal observation).

This study represents the first step towards elucidating the evolution of ant-plant associations in *Triplaris*. In order to understand this association, the interspecific relationships of *Triplaris* were studied using five molecular markers (two chloroplast and three nuclear), and those of the *Pseudomyrmex triplarinus* subgroup with two markers (one mitochondrial and one nuclear). Additionally, a dataset of over 180 collections including all the identified *Triplaris* species with their associate ant colony was compiled and the distribution mapped in ArcGIS. Additional georeferenced collections for *Triplaris* (tropicos.org, GBIF) and associate *Pseudomyrmex* (PS Ward collection) were compiled to model the distribution of both organisms. While most ant-plant interactions have documented specificity between clades but not at the species level, this study shows that some species may have evolved high host specificity.

## Materials and Methods

Permits for field work in Peru were granted by INRENA, for Brazil by CNPq, and for Costa Rica by InBio.

### Sampling and DNA amplification

In this study the phylogenies of both, *Triplaris* and *Pseudomyrmex* were reconstructed using multiple gene regions as well as individuals. Twelve species of *Triplaris* were sampled (of the 18 described; [29, 30]), for a total of 32 individuals. Several individuals per species were included, especially for those that have wide distribution ranges. Only 12 of the 18 described species of *Triplaris* were sampled since several species are highly restricted to remote geographical areas (i.e., *T*. *efistulifera*, *T*. *matogrossensis*, *T*. *moyobambensis*, and *T*. *physocalyx*), and therefore are not easily collected in the field. Herbarium material was available for the six remaining species but amplifications were not successful. Two representatives of *T*. *melaenodendron* were also included, since two subspecies have been recognized (*T*. *melaenodendron* subsp. *melaenodendron* and *T*. *melaenodendron* subsp. *colombiana*; [29]). For the analysis five outgroups were chosen: *Coccoloba swartzii*, *Ruprechtia chiapensis*, *Ruprechtia fusca*, *Ruprechtia tangarana* and *Salta triflora*, based on previous studies by Sanchez and Kron [34].

Total DNA was extracted from silica-dried leaf material when available, or from herbarium material, using the Qiagen DNeasy Mini Plant Kit (Valencia, CA). In the case of herbarium samples, plant material was manually ground and incubated for 18 h with 30 μL of BME (beta-mercapto-ethanol) and 30 μL of proteinase K before continuing with the protocol for the DNeasy kit, with a final elution of 100 μL. In addition, extracted DNA aliquots were obtained through the generosity of Toby Pennington (Royal Botanic Garden, Edinburgh), and Janelle Burke (Cornell University). Data for the reconstruction of the plant phylogeny includes two non-coding chloroplast regions: *psbA-trnH*, *rps16-trnK*, and three nuclear regions: nrITS, the second intron of the low-copy nuclear region *Leafy* (*lfy2i*) and the third intron of the nitrate reductase gene NIA (NIA3i). Protocols for standard polymerase chain reaction (PCR) followed those of Sanchez and Kron [35]. Primers for *psbA-trnH* and *rps16-trnK* were derived from Shaw et al. [36]. Primers for *lfy2i* are described in Sanchez and Kron [34] and primers for NIA3i were derived from Howarth and Baum [37]. PCR products were cleaned using Qiagen QIAquick PCR purification columns (Valencia, CA). All sequences were run on an ABI 377 Automated DNA Sequencer (Ramsey, MN) at Wake Forest University’s Automated DNA Sequencing Facility. Sequences were edited with Sequencher v.3.1.1 (Ann Arbor, MI). In the case of ITS and chloroplast DNA regions, PCR product purification was followed by direct sequencing. For *lfy2i* and NIA3i, purification was followed by cloning using the Invitrogen TOPO TA Cloning kit (Carlsbad, CA). Cloning products were cleaned with ExoSAP-IT (Affymetrix Inc, CA).

A total of 22 species of *Pseudomyrmex* were sampled for this study. Most of the sequences derive from GenBank (S2 Table), except for those belonging to the *Pseudomyrmex* associated with *Triplaris*. A total of eight species and 15 individuals were included to represent the *P*. *triplarinus* subgroup as well as other species of *Pseudomyrmex* that can colonize *Triplaris*. Of the eight ant species collected in *Triplaris*, four are considered specialists (*P*. *dendroicus*, *P*. *mordax*, *P*. *triplaridis*, and *P*. *triplarinus*) and four are considered generalists (i.e., can inhabit other plant species; *P*. *elongatus*, *P*. *gebellii*, *P*. *longior*, and *P*. *viduus*). All ant identifications were provided by P. Ward (UC Davis, CA). Total DNA was extracted from ants preserved in 90% ethanol, using the Qiagen DNeasy Tissue Kit (Valencia, CA) and following the instructions by the manufacturer. Protocols for PCR followed those of Kautz et al. [38]. Data for the phylogenetic reconstruction of the ant phylogeny includes a fragment covering most of the mitochondrial cytochrome oxidase one gene, COI, and the nuclear long-wavelength rhodopsin gene (LR). Primers for LR were derived from Ward and Downie [39] and for COI two sets of primers (COI-LCO and COI-HCO; COI-Jerry and COI-Pat) were used following those published by Kautz et al. [38]. PCR products were cleaned using the Macherey-Nagle NucleoFast 96-well plate (Bethlehem, PA), and sequenced using Big Dye chemistry with an ABI 3730 automated sequencer (PE Applied Biosystems, Foster City, CA) at Duke University.

### Sequence alignment and phylogenetic analyses

All sequences were aligned using Muscle [40] and subsequently adjusted by hand in Mesquite version 2.75 [41], as needed. All matrices are available in TreeBASE (study number S18218) and all sequences are deposited in GenBank (S1 and S2 Tables). Insertions/deletions were not coded as characters in this analysis.

The gene regions for both plants and ants were initially analyzed separately, then concatenated and arranged into partitions corresponding to the different loci (Table 1). Before the analyses, the dataset matrices for each organism were tested for incongruence, using the incongruence length difference (ILD) test as implemented in PAUP* version 4.0b1.0 [42]. All ILD tests used default parameters and 1000 replicates. The ILD test is often used to test if different molecular regions are or not congruent, although it is known to be sensitive to between-partition differences in evolutionary rates and extremes of rate heterogeneity among sites within the data [43, 44, 45, 46]. Therefore, many studies have currently suggested that even if the ILD test shows significant incongruence, concatenating datasets from different regions may not result in misleading phylogenies (e.g., [44, 45, 46]. In this case, when there was significant incongruence (*p* = 0.01) between regions, support for conflicting topologies of the analysis was assessed, before deciding whether to combine. The concatenated matrix for *Triplaris* and for *Pseudomyrmex* was analyzed using the program PartitionFinder v.1.1.1 [47], in order to find the best-fitting combination of partitions and models of sequence evolution for the concatenated nucleotide alignment (Table 2).

**Table 1:** Statistics for the gene regions used for *Triplaris* and *Pseudomyrmex*.

Analysis	Statistic						Combined
		*psbA-trnH*	*rps16-trnK*	ITS	*lfy2i*	NIA3i	
*Triplaris*	Length	1042	992	786	1662	1376	5858
	Var. sites (%)	373 (35.8)	152 (15.3)	254 (32.3)	473 (28.5)	542 (39.4)	1794 (30.6)
	PIC (%)	108 (10.4)	55 (5.5)	94 (12)	141 (8.5)	158 (11.5)	556 (9.5)
	Miss. taxa (%)	4 (10.8)	2 (5.4)	2 (5.4)	9 (24.3)	11 (29.7)	28 (15.1)
		COI	LR				
*Pseudomyrmex*	Length	1528	597				2125
	Var. sites (%)	621 (40.6)	167 (27.8)				788 (37.1)
	PIC (%)	572 (37.4)	101 (16.9)				712 (33.5)
	Miss. taxa (%)	4 (12.1)	4 (12.1)				8 (12.1)

Length = aligned length; PIC = parsimony informative characters.

**Table 2:** Partitions and models for Maximum Likelihood (ML) and Bayesian inference based on PartitionFinder.

		PARTITIONS				
		*psbA-trnH*	*rps16-trnK*	ITS	*lfy2i*	NIA3i
*Triplaris*	Model ML	GTR+G	GTR+I+G	GTR+I+G	GTR+I+G	GTR+I+G
	Model Bayesian	F81+G	GTR+I+G	GTR+I+G	HKY+I+G	HKY+I+G
		COI	LR			
*Pseudomyrmex*	Model ML	GTR+I+G	GTR+G			
	Model Bayesian	GTR+I+G	K80+G			

Maximum Likelihood (ML) analyses and Bayesian inference were conducted on the partitioned matrices of the plants (*Triplaris*) and the ants (*Pseudomyrmex*). For ML, the PartitionFinder configuration file comprised of: branch lengths linked across partitions, the corrected Akaike Information Criterion (AICc) used for comparing models and partition schemes, and data blocks corresponding to the different genes, analyzing all possible partition schemes. ML analyses were conducted on RAxML 7.2 [48] under a partition model (Table 2) and 1,000 bootstraps replicates using the rapid RAxML bootstrapping algorithm. For Bayesian inference, the configuration file of PartitionFinder comprised of branch lengths linked across partition, set of models restricted to those available in MRBayes, Bayesian Information Criterion (BIC) used for comparing models and partition schemes, and data blocks corresponding to the different genes, analyzing all possible partition schemes (Table 2). The concatenated dataset was analyzed using Bayesian phylogenetic reconstruction in MrBayes v3.2.5 [49], with the preferred partitions and models of sequence evolution (Table 2). Parameters were unlinked to allow the different partitions to evolve under different rates. The Markov Chain Monte Carlo (MCMC) algorithm was run for 100 million generations, starting with a random tree and sampling every 10,000 generations. Two independent analyses of two chains each for the MCMC algorithm were run, and the temperature parameter was adjusted to 0.025 to keep appropriate heat and acceptance rates ranges. Branch lengths of the trees were saved. The first 10 million generations were discarded (i.e. treated as “burn-in”) after assessment in the software Tracer v1.6 [50], and the posterior probability (PP) values were estimated from the remaining trees by computing a 50% majority-rule-consensus tree. Trees were visualized using FigTree v1.4.2 (http://tree.bio.ed.ac.uk/software/figtree/). It is important to note that RAxML only supports the GTR nucleotide substitution model. Therefore, the choice of model of ML and Bayesian PartitionFinder is often different (Table 2).

### Ant-plant interactions

For studying the interaction between plants and ants, the simplified phylogenies of both organisms were compared with a tanglegram. Tanglegrams are often used to compare evolutionary histories of host and parasite species and to analyze genes of species in the same geographic area [51]. Additionally, all of the available collections (a total of 186 collections) from A Sanchez, PS Ward, and the literature [23, 33, 52] were compiled with information of the plant host species and its associate ant colony (S3 Table), and mapped using ArcGIS (Esri, Redlands, CA). These collections include specimens from Bolivia, Brazil, Colombia, Costa Rica, Ecuador, Guyana, Panama, Peru, and Venezuela.

In addition, a total of 1876 georeferenced collections of *Triplaris* (including 783 collections from tropicos.org and 907 from GBIF.org: http://doi.org/10.15468/dl.ziz5x7), and 739 collections of *Pseudomyrmex triplarinus* subgroup (an additional 594 collections kindly provided by PS Ward), were compiled in order to model the geographic distributions of both organisms using DIVA-GIS v7.5 [53] under the BIOCLIM approach. BIOCLIM [54] is a commonly used modeling program that relates climatic parameters (i.e. temperature, precipitation, etc.) to species presence data to predict areas within which an organism can survive [55]. Climate data was obtained from WorldClim, v1.4 ([56], http://www.worldclim.org) at 30 arc-second (approximately 1 km2 grid cells) resolution, which represents monthly precipitation and temperature data of the period 1950–2000. The model uses observed point distributions (specimen data) and spatially continuous environmental data (minimum and maximum values a species occurs at for each climatic variable within the model) to map species occurrences and to predict areas that have not been sampled but where the species might be found based on its ecological niche. Finally, areas of suitable habitat that overlap between the ranges of plants and ants were mapped to identify which locations are more important for the mutualism.

## Results

### *Triplaris*

**Fig 2:**
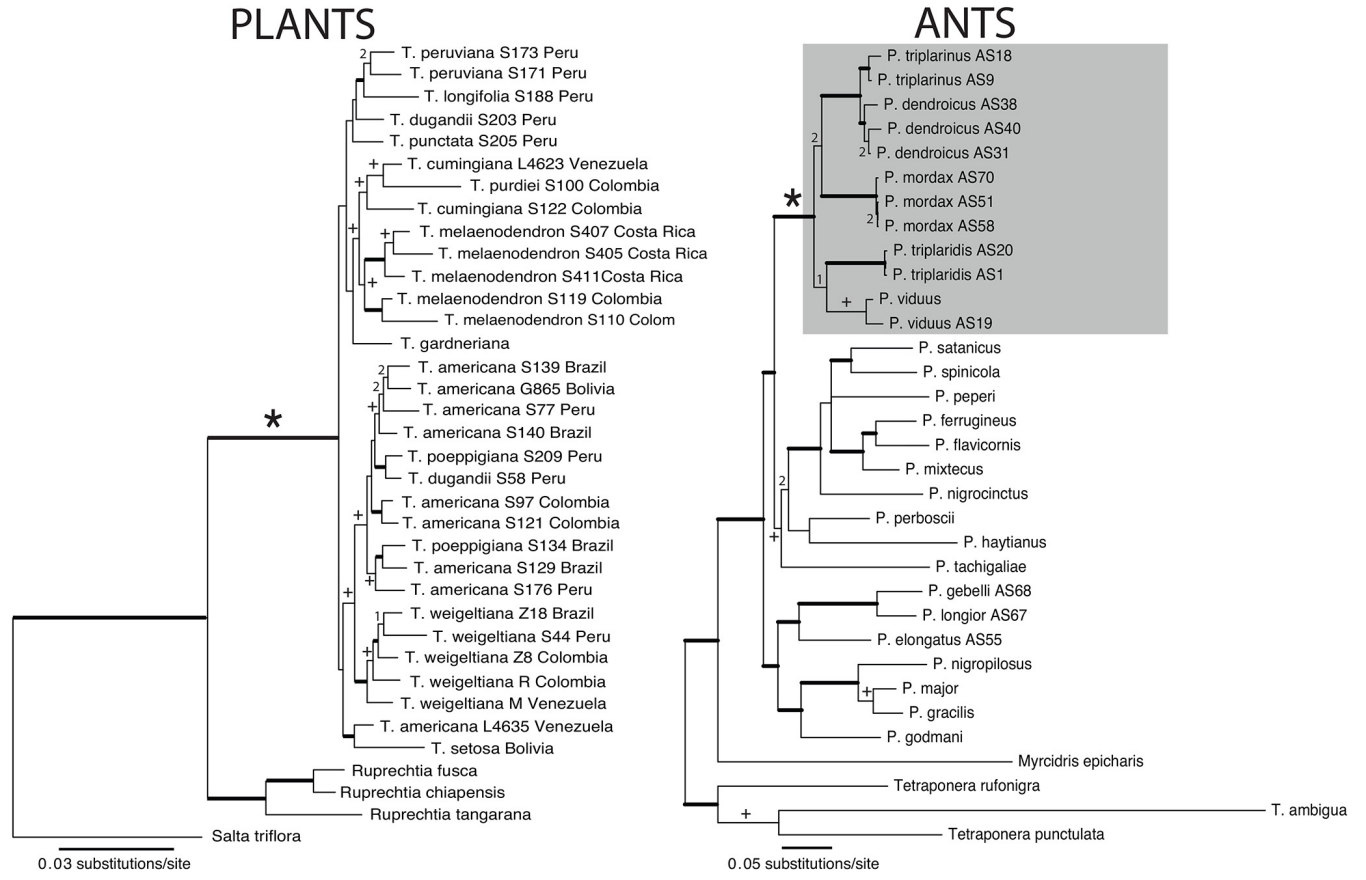
Phylograms obtained from Bayesian inference for the interspecific relationships of *Triplaris* and of *Pseudomyrmex* with emphasis on the *P*. *triplarinus* subgroup (denoted by an asterisk). Thick lines represent branches supported by posterior probabilities (PP) > 0.95 and Maximum Likelihood (ML) bootstrap values > 90%. Cross symbols indicate PP values between 0.9–0.95 and ML bootstraps between 60% and 90%. A number (1) represents a topology only supported by Bayesian analyses (PP > 0.9) and a (2) a topology supported by ML (bootstrap > 60%). The four obligate symbiont species of ants are highlighted in grey. For collection information see S1 and S2 Tables.

Table 1 presents a complete list of gene regions and combined data matrix statistics. For these analyses 32 individuals from 12 species of *Triplaris* were sampled. Several accessions for species that have a wide geographic distribution were included: nine accessions of *T*. *americana* (the most widespread species), five individuals of both *T*. *melaenodendron* and *T*. *weigeltiana*, and two of each, *T*. *cumingiana*, *T*. *dugandii*, *T*. *peruviana*, and *T*. *poeppigiana* (Fig 2). For all other species only one accession was included, since those species have a restricted distribution.

The most variable region was ITS (12% PIC) and the least *rps16-trnK* (5.5% PIC; Table 1). Although there was incongruence between chloroplast and nuclear regions according to ILD (p = 0.01), when bootstrap supports between regions were compared, however, few relationships that were incongruent are moderately or highly supported (> 70%). In general, few interspecific relationships of *Triplaris* were supported with the individual gene regions (three clades for the chloroplast data set, five for ITS, seven for *lfy2i*, and four for NIA3i) and there were only three supported incongruences. The first incongruence was within the clade comprising *T*. *purdiei* and the two accessions of *T*. *cumingiana*, the second in the reconstruction of relationships within *Ruprechtia* and third, the placement of *Salta*. The decision to combine datasets was made since only one incongruence concerned *Triplaris*. The total combined matrix had 9.5% PIC (Table 1). *Triplaris* received strong support as monophyletic in Bayesian and ML analyses (PP: 1, ML: 100%; Fig 2) and the analyses were congruent.

Three supported clades correspond to currently recognized species: *T*. *melaenodendron* (PP 0.93, ML 65%), *T*. *peruviana* (56%), and *T*. *weigeltiana* (1, 96%). Other species such as *T*. *americana*, *T*. *cumingiana*, *T*. *dugandii*, and *T*. *poeppigiana*, were not recovered as monophyletic (Fig 2). However, when the individual accessions are examined closely, there is evidence for some geographic structure. Some clades correspond to individuals collected in sympatry, such as the case of *T*. *poeppigiana* S134 and *T*. *americana* S129 (Acre, Brazil; 1, 91%), *T*. *dugandii* S58 and *T*. *poeppigiana* S209 (Loreto, Peru; 0.99, 84%), and the accessions of *T*. *americana* collected in Colombia (S97 and S121: 1, 97%). *Triplaris purdiei* is nested within *T*. *cumingiana* and the clade is moderately supported (0.95, 86%).

### *Pseudomyrmex*

When the matrices were analyzed separately, COI was more parsimony informative than LR (Table 1). Both regions were incongruent according to ILD (p = 0.01), but the reconstructions differed in the interspecific relationships of *Pseudomyrmex* not associated with *Triplaris* and relationships within the outgroup (*Tetraponera*). The eight species of *Pseudomyrmex* collected from *Triplaris* did not present supported incongruences between data sets. Since the primary interest was in recovering the relationships of the *Pseudomyrmex* associated with *Triplaris*, the decision was to combine all data sets, maintaining the individual partitions. As a result, the total combined matrix had 2125 bp and 33.5% PIC (Table 1). For both analyses (Bayesian and ML), five of the eight ant species collected in *Triplaris* were recovered in a strongly supported clade (Fig 2; PP: 1, ML: 100%), four of which are considered obligate symbionts. *Pseudomyrmex dendroicus* and *P*. *triplarinus* are strongly supported as sister (1, 98%) and they are in turn sister to *P*. *mordax* (60% bootstrap). The placement of *P*. *viduus* and *P*. *triplaridis* was not resolved under ML (< 50%), but Bayesian placed them as sister to each other (0.98).

### Ant-plant interactions

In order to understand the ant-plant interaction between *Triplaris* and *Pseudomyrmex triplarinus* subgroup (sensu Ward) all of the available collections of *Triplaris* and their resident colony were compiled and compared. From a total of 186 collections, 134 correspond to obligate symbionts (72.8%), 37 were inhabited by other ant species (20.1%) and 15 had no ants (8.1%).

**Fig 3:**
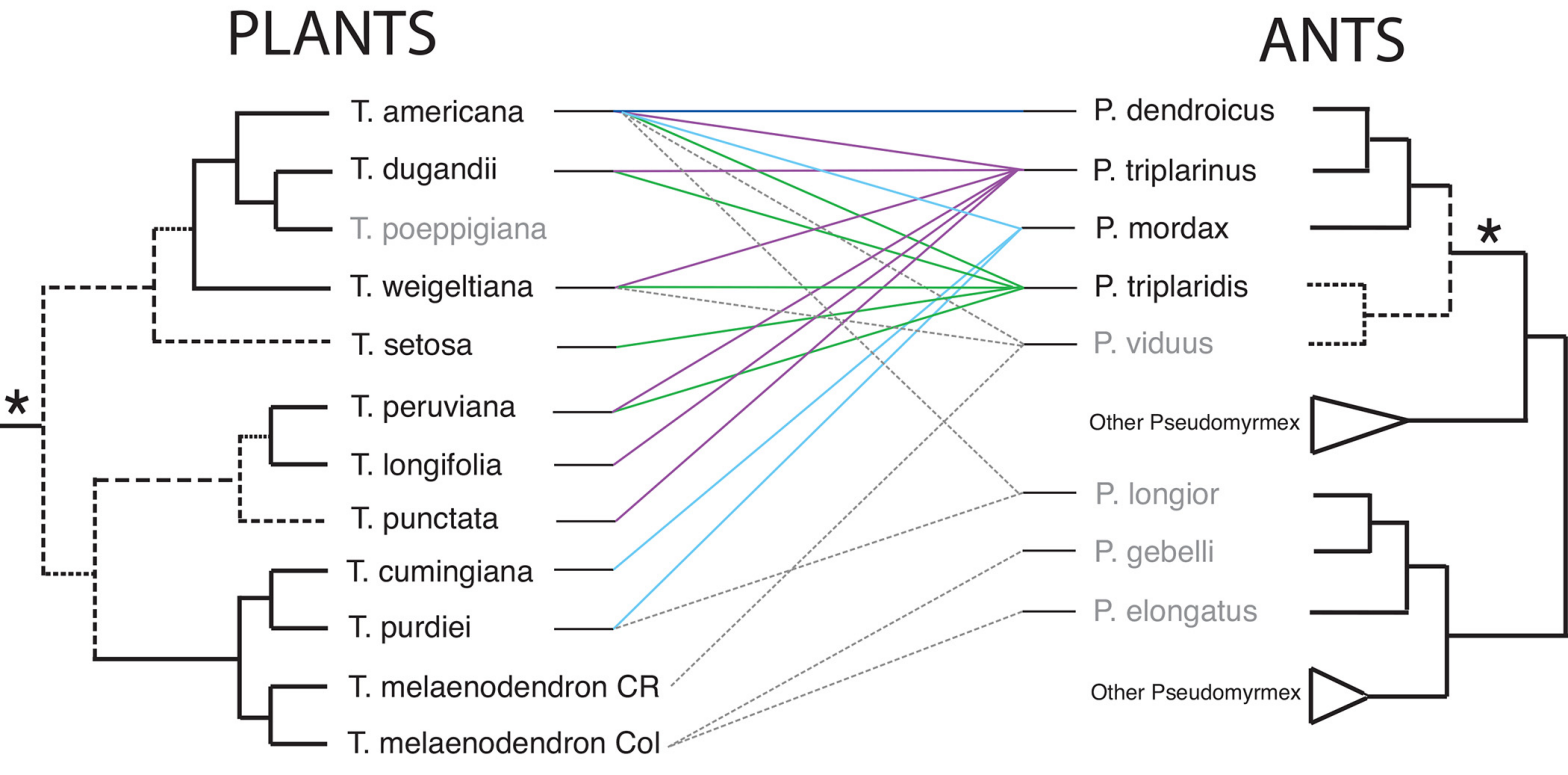
Tanglegram comparing a reduced phylogeny of *Triplaris* and of the *Pseudomyrmex triplarinus* subgroup (denoted by an asterisk). Colored lines between trees indicate associations reported in the literature, and information derived from Sanchez and Ward collections (same color key as in S3 Table). Dashed lines indicate associations with generalist ants (in grey). Note that *T*. *melaenodendron* only associates with generalist ants and *Triplaris poeppigiana* was only found in association with ants of the genus *Azteca* (Table 3). Nodes with less than 0.9 PP or 50% bootstrap (based on Fig 2) are denoted by dashed branches.

Overall the relationships between *Triplaris* and *Pseudomyrmex triplarinus* subgroup are promiscuous (Fig 3); for most species there is no consistent pattern of specialization since one species of plant can associate with multiple species of ants and vice versa. Plants with a wide geographic distribution, such as *T*. *americana* are inhabited by four obligate species of *Pseudomyrmex* as well as other ants (Fig 3), while species such as *T*. *longifolia* were only collected in association with *P*. *triplarinus* (Table 3, Fig 3). Species such as *T*. *cumingiana* and *T*. *melaenodendron* (from Colombia and Costa Rica) were mostly found with non-specific ants (Fig 3). In the case of *T*. *poeppigiana*, no association with *Pseudomyrmex* was found, but instead with ants of the genus *Azteca* (Table 3). For other plant species such as *T*. *punctata* and *T*. *setosa*, there is only one ant colony record at this time (Table 3), making it difficult to understand the degree of specificity of the interaction. With the exception of *T*. *cumingiana and T*. *melaenodendron* (60% not colonized), the percentage of ant occupancy is high. *Triplaris americana*, *T*. *longifolia*, *T*. *peruviana* and *T*. *purdiei* were always found in association with ants, and *T*. *weigeltiana* only had a 2.3% of individuals with no ant colony (Table 3).

**Table 3:** Percentage of occupancy for *Triplaris* species based on 184 collections (S3 Table).

Plant species	Ant colony	% occupancy
*T*. *americana*	*P*. *dendroicus*	49.3
N = 75	*P*. *mordax*	16
	*P*. *triplaridis*	2.7
	*P*. *triplarinus*	26.7
	*P*. *viduus*	1.3
	*P*. *elongatus*	1.3
	*Azteca* sp.	1.3
	*Crematogaster* sp.	1.3
*T*. *cumingiana*	*P*. *mordax*	20
N = 5	*Crematogaster* sp.	20
	No Ants	60
*T*. *dugandii*	*P*. *triplaridis*	46.2
N = 13	*P*. *triplarinus*	30.8
	*P*. *ultrix*	7.7
	No Ants	15.4
*T*. *longifolia* N = 5	*P*. *triplarinus*	100
*T*. *melaenodendron*	*P*. *gebelli*	10
Colombia	*P*. *longior*	10
N = 10	*Crematogaster* sp.	20
	No Ants	60
*T*. *melaenodendron*	*P*. *viduus*	66.7
Costa Rica	*Crematogaster* sp.	11.1
N = 9	No Ants	22.2
*T*. *peruviana* N = 9	*P*. *triplaridis*	55.6
	*P*. *triplarinus*	33.3
	*Azteca* sp.	11.1
*T*. *poeppigiana*	*Azteca* sp.	90
N = 10	No Ants	10
*T*. *punctata* N = 1	*P*. *triplarinus*	100
*T*. *purdiei*	*P*. *mordax*	25
N = 4	*P*. *elongatus*	25
	*Crematogaster* sp.	50
*T*. *setosa* N = 1	*P*. *triplaridis*	100
*T*. *weigeltiana*	*P*. *triplaridis*	54.5
N = 44	*P*. *triplarinus*	2.3
	*P*. *viduus*	6.8
	*Azteca* sp.	18.2
	*Crematogaster* sp.	9.1
	Other genera*	6.8
	No Ants	2.3

* *Camponotus sexguttatus, Cephalotes ramiphilus, Dolychoderus bidens.*

**Fig 4:**
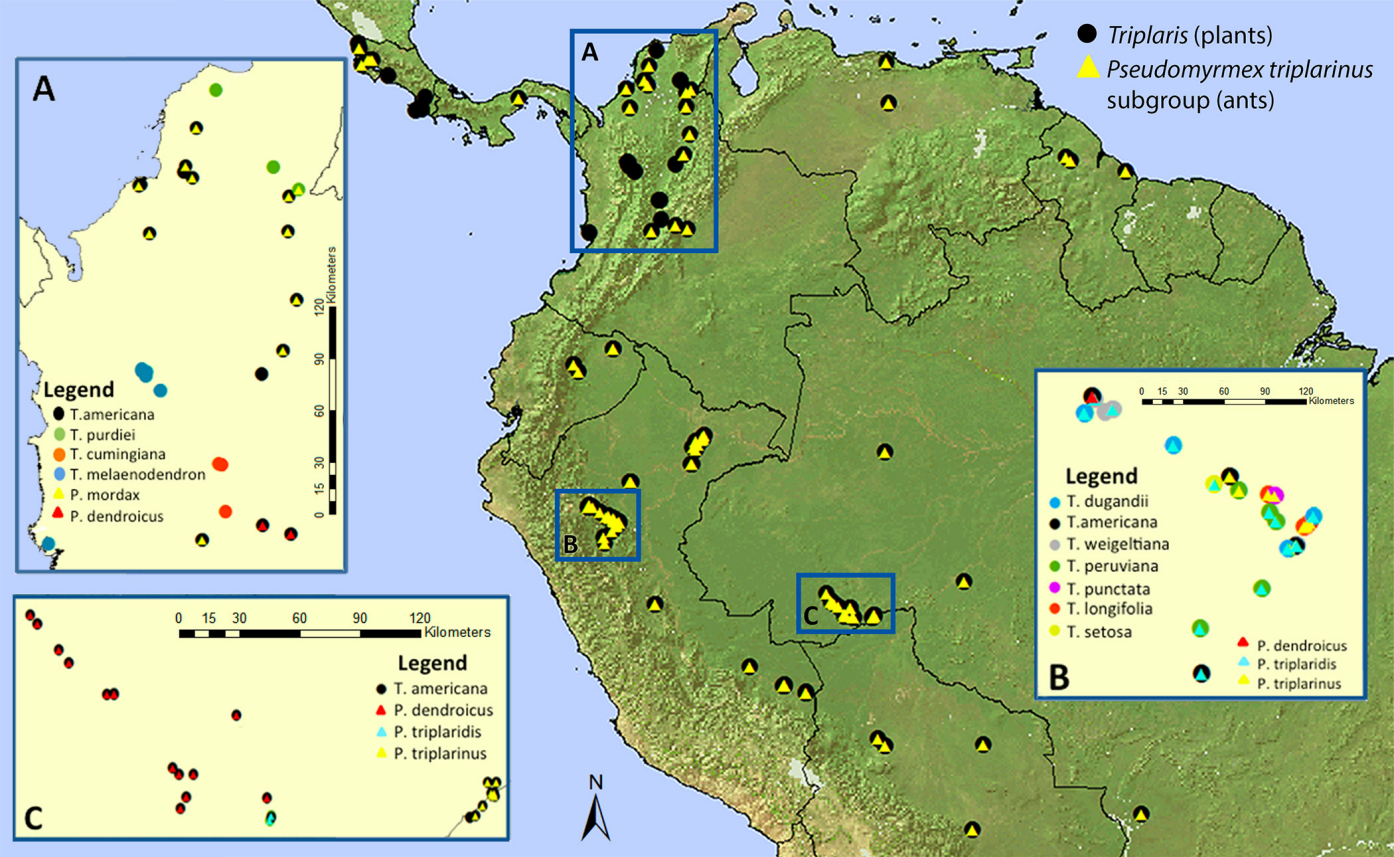
Geographical distribution of all *Triplaris* and associate obligate *Pseudomyrmex* ant collections known to date (S3 Table). Circles represent plant species and triangles ant species. Inserts A to C are zoom-in areas, where several species occur in sympatry. A. Central and northern Colombia. B. San Martin, Peru. C. Acre, Brazil. Colors represent different species, see legend insert.

Ants that have a more extensive distribution associate with more than five species of *Triplaris*: *P*. *triplaridis* and *P*. *triplarinus* are associated with multiple host plants growing in sympatry (Fig 4). However, based on 37 collections of *P*. *dendroicus* from five different countries (S3 Table), there was faithfulness to a single host. These ants have only been collected in plants of *T*. *americana* (Fig 3) even when other *Triplaris* species occur in sympatry (i.e. *T*. *dugandii*, *T*. *longifolia*, *T*. *peruviana*, *T*. *weigeltiana*; Fig 4B). *Triplaris americana* has a more ample range of distribution than *P*. *dendroicus* (S1 Fig), but both organisms overlap in several regions of their range in Ecuador, Colombia, Peru, northern Bolivia, and western Venezuela and Brazil.

**Fig 5:**
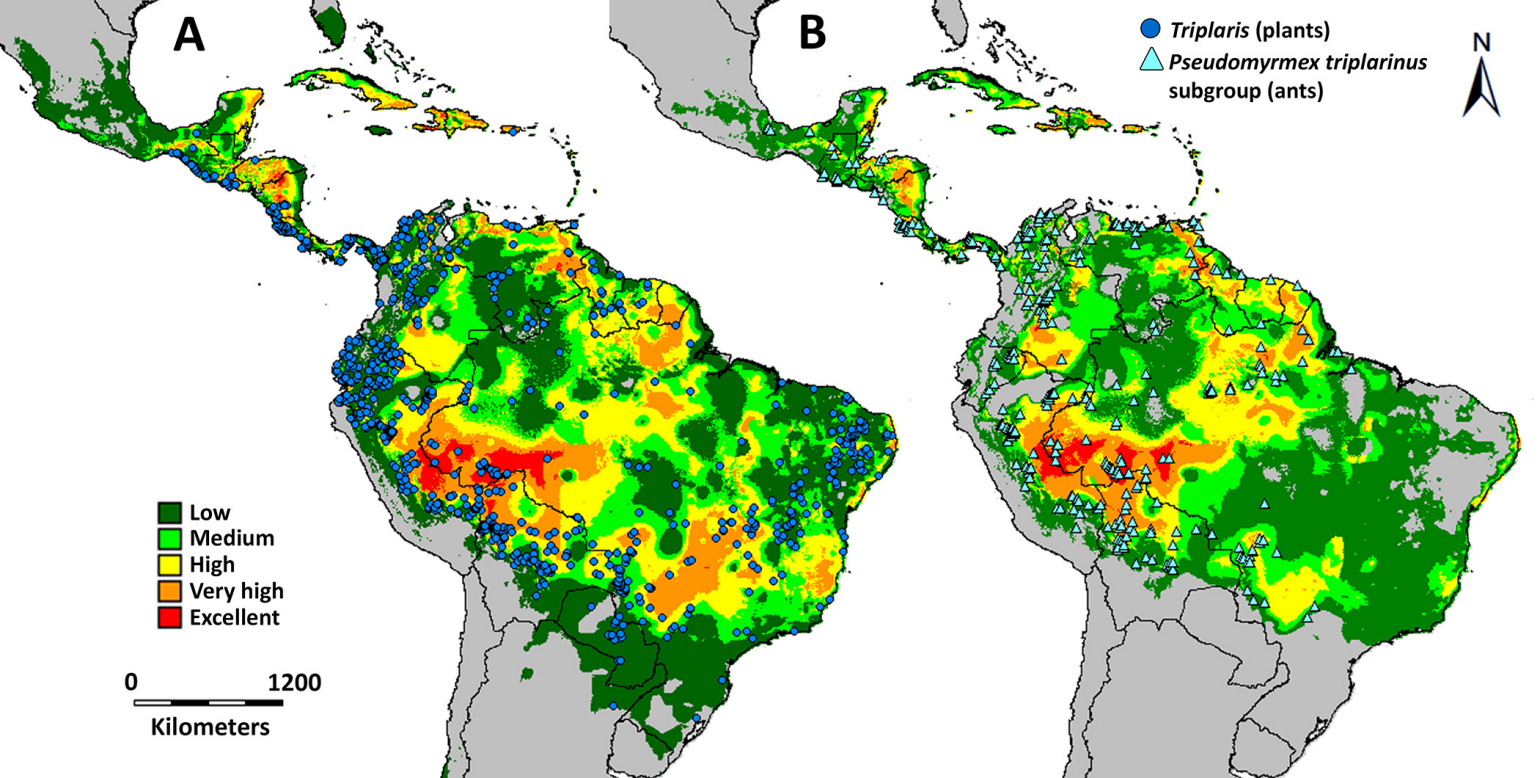
Range of distribution for *Triplaris* (A) and *Pseudomyrmex triplarinus* subgroup (B) as predicted by DIVA-GIS. A. The circles represent the 1876 collections of *Triplaris*; B. Triangles indicate the distribution of the 739 collections of the obligate *Pseudomyrmex*. In both maps, warmer colors (i.e. orange and red) indicate the areas where the climatic conditions are most suitable for the occurrence of both organisms. Grey areas indicate unsuitable climatic conditions.

The geographic distributions of *Triplaris* and *Pseudomyrmex triplarinus* subgroup were modeled using 1876 and 739 georeferenced collections, respectively (Fig 5). In general, the range of distribution of *Triplaris* is larger than its obligate associates. *Triplaris* can extend further to the south (into Paraguay and Northern Argentina), to the north (Mexico) and to the east (eastern Brazil). Both, plants and ants tend not to be found in dry areas and in the higher altitudes of the Andes, but ants seem more sensitive to these conditions than the plants, given their more restricted distribution (Fig 5).

**Fig 6:**
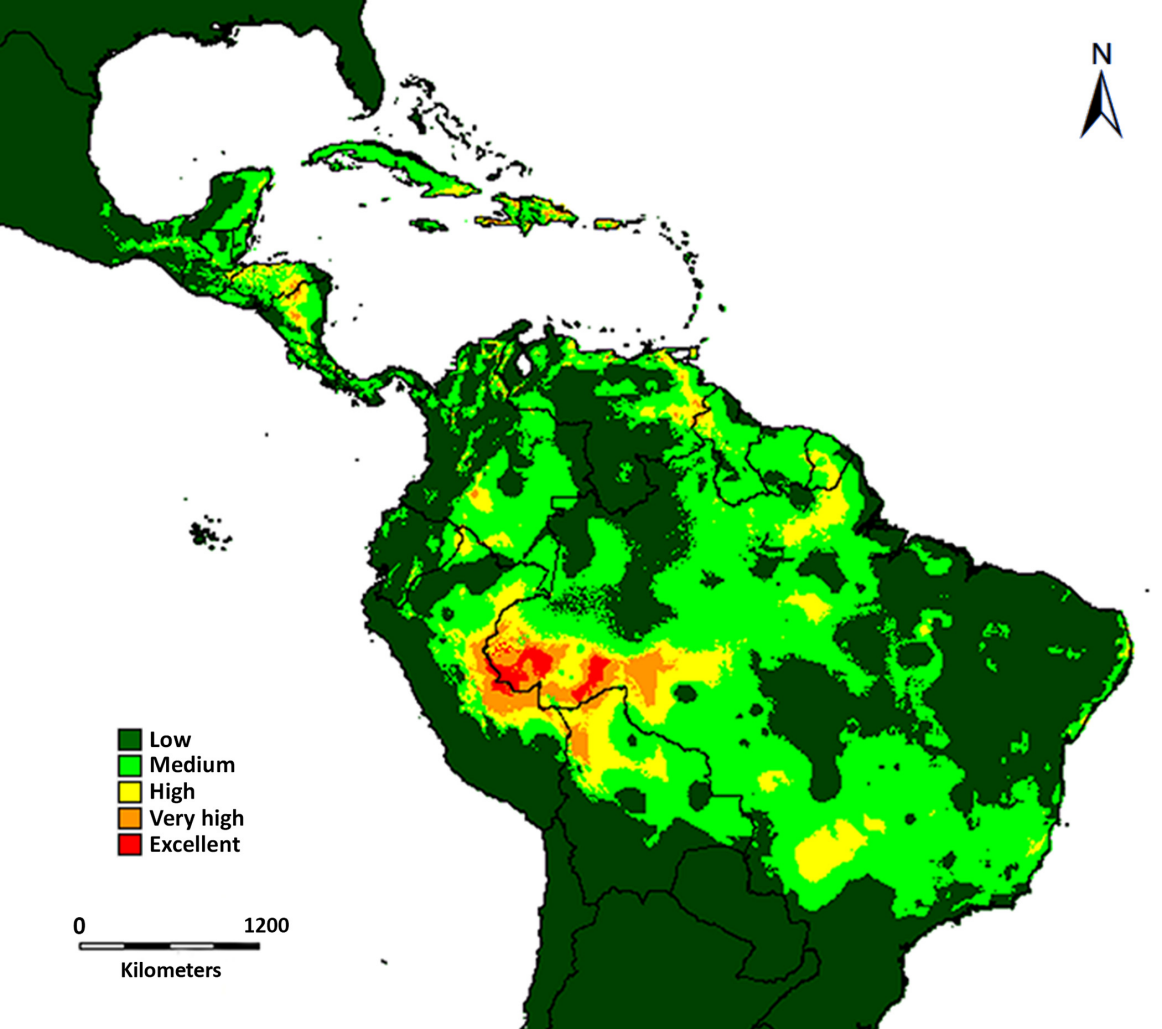
Range overlap between *Triplaris* and its obligate associate ants as predicted by DIVA-GIS. Warmer colors (i.e. orange and red) indicate increased likelihood of co-occurrence.

When both species’ ranges of distribution are overlapped, the western portion of the Amazon Basin shows the highest likelihood of co-occurrence (Fig 6). This area comprises parts of eastern Peru (southern Loreto, Ucayali and Madre de Dios), western Brazil (Acre, Amazonas, Mato Grosso, and Rondônia), and northern Bolivia (Pando) (Fig 6). Some other areas in southern Colombia, eastern Venezuela, French Guiana, Brazil (Pará, Mato Grosso do Sul), northern Nicaragua, and southern Honduras also show high degrees of overlap (Fig 6). Areas such as the Andes Cordillera, Chocó, the Venezuelan savannahs, and eastern Brazil have lower likelihood of co-occurrence.

## Discussion

This is the first phylogenetic study to explore the interspecific relationships of *Triplaris*, the interspecific relationships of the *Pseudomyrmex* associated with *Triplaris*, and to study the interaction using molecular and geographic data (including all the available ant-plant field collections, and museum and herbaria collections). *Triplaris* is monophyletic as well as the *Pseudomyrmex triplarinus* subgroup (Fig 2). The mutualism is mostly promiscuous, with the exception of *P*. *dendroicus’* faithfulness (Fig 3).

*Triplaris* is a monophyletic genus and all of the species are known to be myrmecophytes. In this study, 12 of the 18 species of *Triplaris* were included in a multiple individual analysis. Some species such as *T*. *melaenodendron*, *T*. *peruviana* and *T*. *weigeltiana* were supported as monophyletic, while species such as *T*. *dugandii*, *T*. *poeppigiana* and *T*. *americana* were not (Fig 2). In the case of species with widespread geographical ranges, such as *T*. *americana*, there may be clades that represent reciprocal gene flow due to geographic proximity, since the two accessions of *T*. *americana* from Colombia are more closely related, as are the four accessions from Southeastern Peru, Western Brazil and Bolivia (Fig 2). However, more studies are necessary for understanding the interspecific relationships of *Triplaris*, including the possibility of hybridization and support for the geographical structure seen in this study. The last comprehensive revision of the genus was done by Brandbyge [29].

*Pseudomyrmex triplarinus* subgroup is also monophyletic and with the exception of *P*. *viduus*, the clade is an exclusive associate to *Triplaris*. The *Pseudomyrmex triplarinus* subgroup is confined to South America and Panama with the exception of *P*. *viduus*, which has a widespread distribution, ranging from Mexico to Bolivia and Brazil. Three species have restricted ranges of distribution (*P*. *mordax*, *P*. *vitabilis*, and *P*. *ultrix*), while the other three species (*P*. *dendroicus*, *P*. *triplaridis*, and *P*. *triplarinus*) occur in sympatry and overlap extensively in the Amazon basin (Fig 4, [28]). Within the subgroup, there are two clades, one comprising *P*. *triplaridis* and *P*. *viduus* and another comprising *P*. *mordax*, *P*. *dendroicus*, and *P*. *triplarinus* (Fig 2). These clades are in accordance with Ward [28]. Several other species of *Pseudomyrmex*, outside the *P*. *triplarinus* subgroup, can also colonize the hollow stems of *Triplaris* (i.e., *P*. *elongatus*, *P*. *gebellii*, and *P*. *longior*. Table 3, Fig 3; [28]). These associations have evolved independently from the *P*. *triplarinus* subgroup (Figs 2 and 3) and all of these species are considered generalists since they inhabit several different plant genera and in some cases, even dead twigs (i.e., *P*. *elongatus*).

*Triplaris* and *Pseudomyrmex triplarinus* subgroup overlap in most of their range of distribution (Figs 5 and 6). However, *Triplaris* has a larger range of distribution and occurs in areas where its obligate symbionts do not (Fig 5). Ants are, in general, sensitive to habitat and climatic changes [4, 7], which may restrict their distribution to avoid drier (western South America, northeastern Brazil) and colder areas (the Andes Cordillera). When the ranges of both organisms are overlapped (Fig 6), there is a hotspot for the association in the western part of the Amazon Basin. In this area, six of the seven species of *Pseudomyrmex triplarinus* subgroup [28, S3 Table] and 12 of the 18 described species of *Triplaris* occur ([29], tropicos.org from the Missouri Botanical Garden, GBIF, S3 Table).

### Ant-plant interaction

Few cases of symbioses involve a significant pattern of co-cladogenesis between organisms, and most of the known cases involve parasites and their hosts [57, 58, 59]. Some other cases that were thought to follow co-cladogenesis, such as the case of figs and their pollinators, have been demonstrated to be less specific than once thought [60, 61, 62]. The ant-plant interactions are no exception. The few studies that have addressed this type of association by comparing the phylogenies of both organisms have arrived at the same conclusion: there is no strict cospeciation between the organisms (e.g., [13, 17, 19]).

The case of *Triplaris* and *Pseudomyrmex* follows a similar scenario and is in accordance with the results of Gómez-Acevedo et al. [19]: the relationships are diffuse (Fig 3). However, when the mutualism is explored at a finer scale, there are several degrees of specialization.

Species such as *T*. *americana* and *T*. *weigeltiana* can associate with more than three different species of *Pseudomyrmex* (Fig 3), as well as other ant genera (Table 3). *Triplaris americana* and *T*. *weigeltiana* have ample ranges of distribution; *T*. *americana* is distributed from southern Panama to southeastern Brazil, while *T*. *weigeltiana* is found in the Amazon basin, Venezuela, Guyana, Surinam and French Guyana [29]. These two species can therefore overlap with several obligate ant species (Fig 4). However a certain degree of specialization is evident, since 49% of the 75 collections of *T*. *americana* were colonized by *P*. *dendroicus* and 54% of the 44 collections of *T*. *weigeltiana* were colonized by *P*. *triplaridis* (Table 3). In other cases, there is little specialization to the *Pseudomyrmex triplarinus* subgroup. Species such as *T*. *melaenodendron*, distributed in Central America and Central Colombia [29], were mostly collected in association with non-specific ants (Fig 3; Table 3). *Triplaris melaenodendron* subsp. *melaenodendron* associates with *P*. *viduus* and several other generalist *Pseudomyrmex* species. In this case, the distribution of the plant species does not match the obligate ant species, which are distributed from Panama to southern Brazil [28]. *Triplaris melaenodendron* subsp. *colombiana* was collected in Antioquia and Valle del Cauca (S3 Table), and although obligate ants have been collected in Antioquia (*P*. *dendroicus*, *P*. *mordax*), there are no reports of these ants in Valle del Cauca (Fig 4). Additional sampling in Central Colombia would allow a better understanding of the ant-plant relationships in this subspecies.

There are other cases where there is no association with the *Pseudomyrmex triplarinus* subgroup. *Triplaris poeppigiana* does not associate with *Pseudomyrmex* but with *Azteca* (Table 3). Although *T poeppigiana* is found in sympatry with as many as seven *Pseudomyrmex-*colonized plant species (e.g., in San Martin, S2 Fig) and has prostoma (small unlignified zones between 6 to 10 mm long by which ants gain entry to the domatia), *T*. *poeppigiana* is still not colonized by these ants. This species presents long, ferrugineous hairs in the twigs and leaves that may impair the movement of larger ants such as *Pseudomyrmex*, enabling other genera, such as *Azteca* to colonize them. However, a recent study suggests that *P*. *triplarinus* can chemically discriminate among *T*. *americana* and *T*. *poeppigiana* [26]. Thus, a different chemical profile could make *T*. *poeppigiana* a less suitable host to the obligate *Pseudomyrmex*.

From the ant perspective, most species are highly promiscuous. Species of ants such as *P*. *mordax* and *P*. *triplarinus* do not seem to discriminate between host species, since *P*. *mordax* colonizes the three species of *Triplaris* that overlap its distribution (Table 3, Fig 4A, S3 Fig) and *P*. *triplarinus* can colonize at least five host species growing in sympatry (Figs 3 and 4B). *Pseudomyrmex triplaridis* may show some specificity towards *T*. *weigeltiana* but it still colonizes four other hosts. However, even when most ant species are promiscuous, one species seems faithful to its host. The 37 collection records for *P*. *dendroicus* (Fig 3, S3 Table) show the same pattern: they were all collected in a *T*. *americana* host. Despite the fact that more plant species are sympatric with *T*. *americana* (Fig 4B), *P*. *dendroicus* has not been collected in any other host. Although this pattern could change as more collections of hosts and their resident ants are available, there is still a high degree of specificity by *P*. *dendroicus*. These two organisms co-occur in most of their range of distribution (S1 Fig), which could have led to ecological sorting. Two less well-known ant species from the *P*. *triplarinus* subgroup, *P*. *ultrix* and *P*. *vitabilis*, were not collected in this study. *Pseudomyrmex vitabilis* has not yet been collected from a plant host (the description is based on a queen [28]) and *P*. *ultrix* is only known from a single locality in Ecuador, on *T*. *dugandii* [28]. More collections are therefore necessary to clarify the degree of specificity of these two ant species to their hosts.

Host selection and colony establishment depend on the foundress queens. Since queens suffer from high mortality due to predation and desiccation [63], effectively recognizing their host plays a crucial moment in the establishment of the association [64]. There are three main hypotheses concerning the species specificity of ant-plant interactions throughout the foundations stage [65]. 1) Interspecific competition: in almost all myrmecophytes, multiple ant queens often colonize different modules of the same plant, which can lead to strong intra- and interspecific competition [4]. Strong competition can potentially be the major factor driving specialization in mutualisms [5] and if competitive interactions among ants are sufficiently strong and constant, ecological sorting could produce predictable patterns of ant-plant associations. Plant and ant lineages may have evolved in concert but some factors such as host-switching, secondary colonization and ecological replacement can modify the associations [4]. 2) Exclusion filters could prevent other ant species from entering and colonizing the host. 3) Host recognition could influence the choice of the founding queens. However, several other factors can also cause a lack of host specificity in a symbiotic relationship. Ants may be more sensitive to habitat than to taxonomic differences among symbiotic partners [7, 24], and/or they could be affected by the demographic and life-history characteristics of the plant and ant populations [2]. It is likely that ecological processes that require no strict cospeciation can therefore maintain the symbiosis. In the case of *Macaranga* it has been found that the association can be sensitive to processes such as geographic or biotic isolation, climatic change and fragmentation that cause local extinctions [66].

In the case of host recognition, ants are known to respond to chemical cues from their hosts. They rapidly recruit in response to herbivory attacks [27, 67] and queens select their host plant primarily based on chemical recognition [64, 65, 68, 69, 70, 71]. Chemical analysis of myrmecophytic *Macaranga* showed that even closely related species, sharing the same ant partners, can have very different scent patterns and that foundress queens have the capacity to distinguish between different hosts [64]. The study also concludes that some queens tend to prefer plants of their ‘usual‘ host-plant spectrum. In addition, a recent study revealed that *P*. *triplarinus* workers are effective in discriminating hosts based on chemical signals and that they are able to discriminate their host (*T*. *americana*) from a closely related species (*T*. *poeppigiana* which, as mentioned before, does not associate with *Pseudomyrmex*) [26]. Even though *P*. *triplarinus* is not as specific as *P*. *dendroicus* (Fig 3), this could explain how queens of *P*. *dendroicus* identify and choose *T*. *americana* based on the chemical cues of the plants they were raised on. Studies using leaf volatiles of different sympatric *Triplaris* species and foundress queens of *P*. *dendroicus* could elucidate if there is strict specificity due to chemical recognition.

Additional sampling of more individuals of *Triplaris* and its associate ants (as well as more gene regions) will improve our understanding of the phylogenetic relationships in both groups and will provide a more complete picture of the degrees of specialization in the different plant species. The present sampling of plants and their resident ant colonies was restricted to some areas of their geographic distribution but did not include extensive sampling in areas such as southern and eastern Brazil, Bolivia, Ecuador, and Venezuela. Therefore, plant species such as *T*. *caracassana*, *T*. *efistulifera*, *T*. *gardneriana*, *T*. *matogrossensis*, *T*. *physocalyx* and *T*. *vestita* were not collected and at present their degree of specialization is not known. Including more plant species and their resident ant colonies is paramount for understanding the dynamics of this mutualism and the evolution of the different degrees of specialization. Although the mutualism is mostly promiscuous, ecological sorting can explain the faithfulness of *P*. *dendroicus* to *T*. *americana*.

## Supporting Information

S1 Fig Distribution of *T*. *americana* and *P*. *dendroicus*. The distribution was based on more than 520 collections of *T*. *americana* (A Sanchez, tropicos.org, GBIF) and 145 collections of *P*. *dendroicus* (PS Ward, A Sanchez).
(TIF)

S2 Fig Distribution of seven *Triplaris* species, with emphasis in Peru. The seven species converge in San Martin, Peru. Colors are the same as in Fig 4B.
(TIF)

S3 Fig Distribution of *P*. *mordax* and its hosts, *T*. *americana*, *T*. *cumingiana* and *T*. *purdiei*. *Pseudomyrmex mordax* occurs in Panama, Colombia and Venezuela (based on 116 collections), where it colonizes the hosts that overlap its range. Colors are the same as in Fig 4A.
(TIF)

S1 Table Voucher information for plant DNA extractions used in this study. Sequences obtained from Genbank are given with their respective site-specific numbers. New sequences generated for this study provide the following information: Taxon, collector(s) and collection number (#), location, herbarium, and Genbank accession numbers. Herbarium acronyms follow Index Herbariorum, E = Royal Botanic Gardens Edinburgh, NY = New York Botanical Garden, BH = Cornell University, MO = Missouri Botanical Garden, WFU = Wake Forest University. NA = not used in this study. In bold are the sequences generated in this study. For more details on collections by Sanchez see S3 Table.
(DOCX)

S2 Table Voucher information for ant DNA extractions used in this study. Sequences obtained from Genbank are given with their respective site-specific numbers. New sequences generated for this study provide the following information: Taxon, collector(s) and collection number (#), location, and Genbank accession numbers. Specimens are part of Sanchez collection with duplicates in UCD (P. Ward collection). NA = not used in this study. In bold are the sequences generated in this study. For more details on collections by Sanchez see S3 Table.
(DOCX)

S3 Table Collections of *Triplaris* and associate ants. # indicates the collection number for each organism. Ant species are color-coded. Plant species in bold indicate individuals associated with obligate ant mutualists. *NI*: indicates collections without information.
(DOCX)
