# Supplementary figures and images for: Fidelity and Promiscuity in an Ant-Plant Mutualism: A Case Study of Triplaris and Pseudomyrmex
Source: PLoS One. 2015 Dec 2;10(12):e0143535. doi: 10.1371/journal.pone.0143535 (PMC4668088; doi:10.1371/journal.pone.0143535)

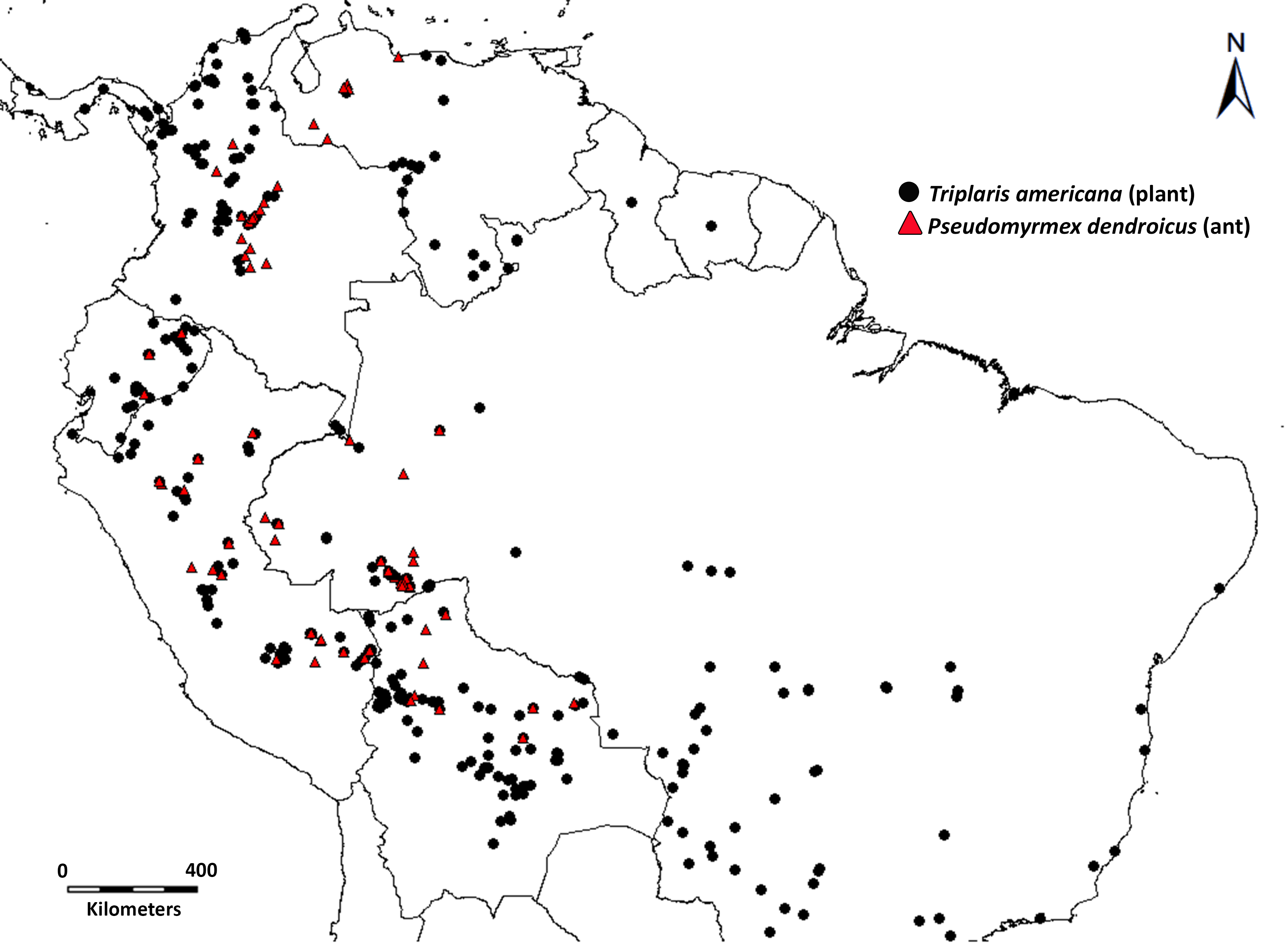

Supplement: S1 Fig — The distribution was based on more than 520 collections of T. americana (A Sanchez, tropicos.org, GBIF) and 145 collections of P. dendroicus (PS Ward, A Sanchez). (TIF) [file pone.0143535.s001.tif]

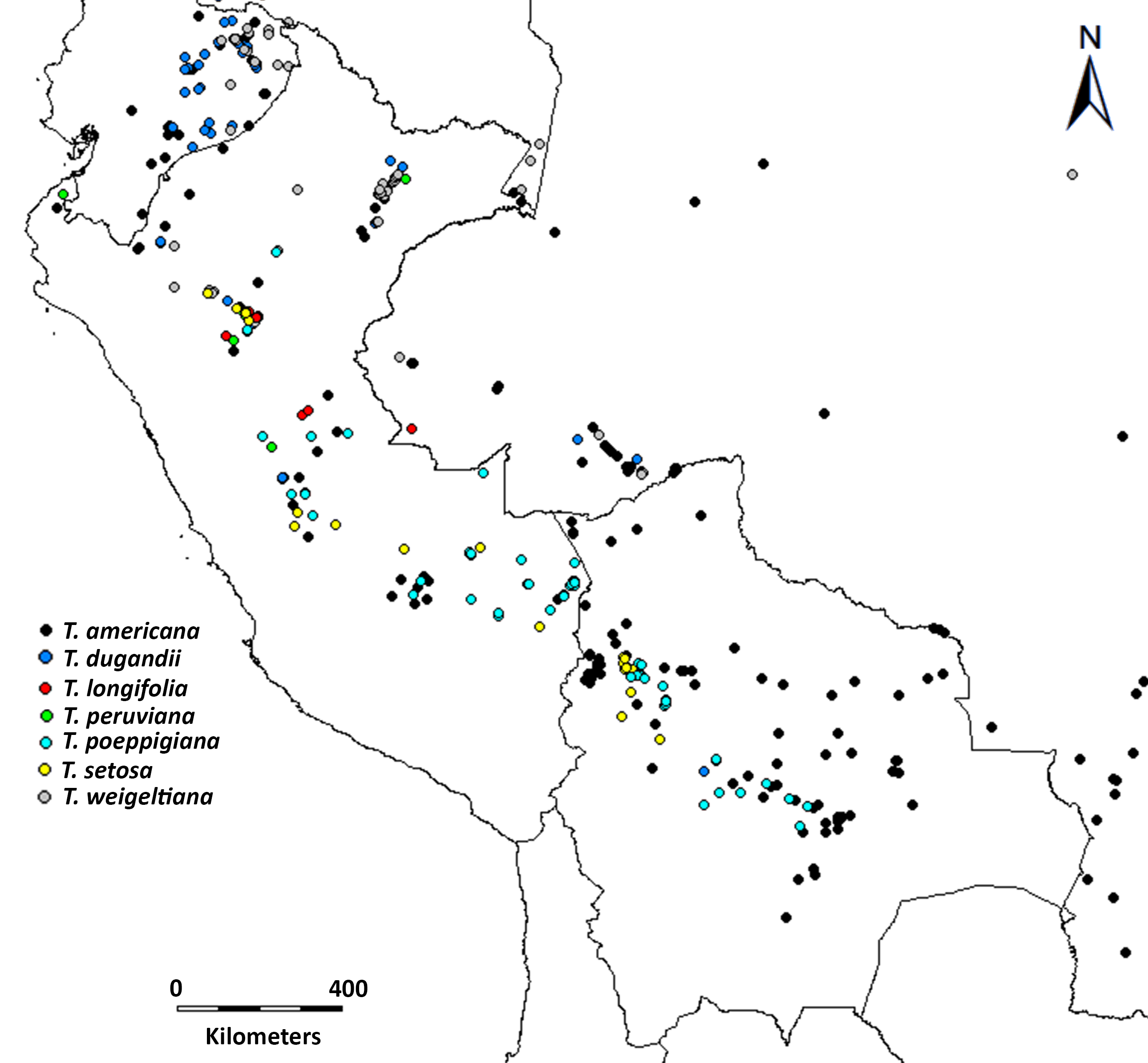

Supplement: S2 Fig — The seven species converge in San Martin, Peru. Colors are the same as in Fig 4B. (TIF) [file pone.0143535.s002.tif]

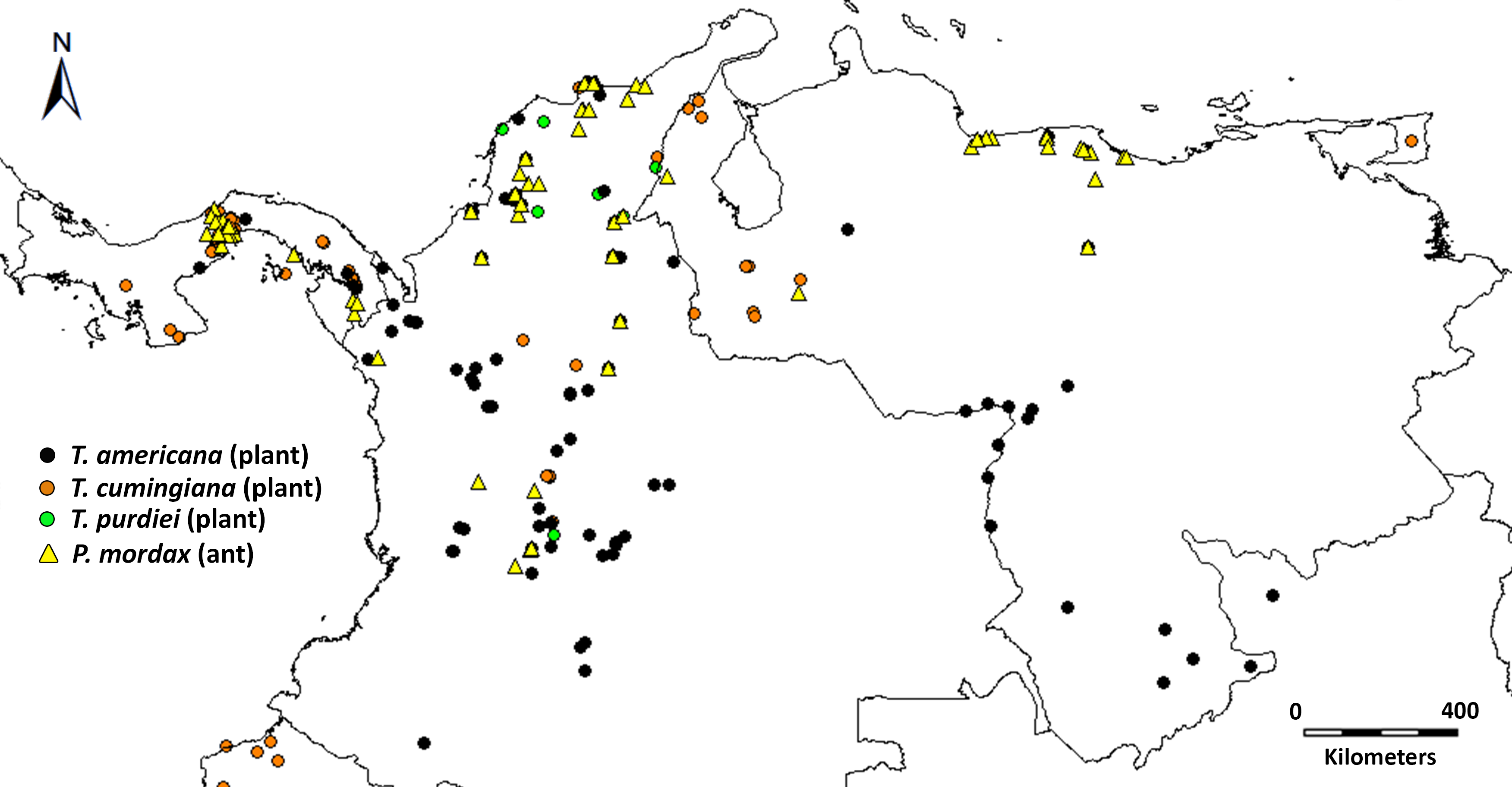

Supplement: S3 Fig — Pseudomyrmex mordax occurs in Panama, Colombia and Venezuela (based on 116 collections), where it colonizes the hosts that overlap its range. Colors are the same as in Fig 4A. (TIF) [file pone.0143535.s003.tif]
