# Supplementary material for: Fidelity and Promiscuity in an Ant-Plant Mutualism: A Case Study of Triplaris and Pseudomyrmex
Source: PLoS One. 2015 Dec 2;10(12):e0143535. doi: 10.1371/journal.pone.0143535 (PMC4668088; doi:10.1371/journal.pone.0143535)
Supplement: S1 Table — Sequences obtained from Genbank are given with their respective site-specific numbers. New sequences generated for this study provide the following information: Taxon, collector(s) and collection number (#), location, herbarium, and Genbank accession numbers. Herbarium acronyms follow Index Herbariorum, E = Royal Botanic Gardens Edinburgh, NY = New York Botanical Garden, BH = Cornell University, MO = Missouri Botanical Garden, WFU = Wake Forest University. NA = not used in this study. In bold are the sequences generated in this study. For more details on collections by Sanchez see S3 Table. (DOCX) [file pone.0143535.s004.docx]

**S1 Table**. Voucher information for plant DNA extractions used in this study. Sequences obtained from Genbank are given with their respective site-specific numbers. New sequences generated for this study provide the following information: Taxon, collector(s) and collection number (#), location, herbarium, and Genbank accession numbers. Herbarium acronyms follow Index Herbariorum, E = Royal Botanic Gardens Edinburgh, NY = New York Botanical Garden, BH= Cornell University, MO = Missouri Botanical Garden, WFU = Wake Forest University. NA= not used in this study. In bold are the sequences generated in this study. For more details on collections by A Sanchez see S3 Table.

| *Species* | *Collector, #* | *Location* | *Herbarium* | *rps16-trnK* | *psbA-trnH* | *ITS* | *Leafy* | *NIA* |
| --- | --- | --- | --- | --- | --- | --- | --- | --- |
| **Outgroup** |  |  |  |  |  |  |  |  |
| *Coccoloba swartzii* Meisn. | A Sanchez 109 | Florida, US-cultivated | WFU | HQ693195 | NA | FJ154469 | EF442787 | **KP236748** |
| *Ruprechtia chiapensis* Lundell ex Standl. & Steyerm. | JM Burke s.n | Yucatan, Mexico | BH | HQ693171 | **KP236809** | FJ154482 | HQ693119 | NA |
| *Ruprechtia fusca* Fernald | CA Pendry 868 | Jalisco, Mexico | E | HQ693177 | **KP236811** | FJ154483 | HQ693122 | **KP236753** |
| *Ruprechtia tangarana* Standl*.* | MR Silman s.n | Madre de Dios, Peru | WFU | HQ693184 | **KP236810** | FJ154485 | HQ693128 | NA |
| *Salta triflora* (Griseb.) Adr. Sanchez | M Nee et al. 53657 | Santa Cruz, Bolivia | MO | HQ693168 | NA | GQ206267 | NA | **KP236756** |
| **Triplaris** |  |  |  |  |  |  |  |  |
| *T. americana* L. | J Gutierrez et al. 865 | Chuquisaca, Bolivia | MO | **KP236787** | **KP236827** | **KP271204** | NA | **KP236758** |
| *T. americana* L. | MA Luckow 4635 | Zulia, Venezuela | BH | HQ693185 | **KP236832** | FJ154486 | HQ693129 | **KP236749** |
| *T. americana* L. | A Sanchez 77 | Madre de Dios, Peru | WFU | **KP236770** | NA | **KP271210** | **KP271222** | **KP236750** |
| *T. americana* L. | A Sanchez 97 | Cesar, Colombia | WFU | **KP236791** | **KP236834** | **KP271195** | **KP271233** | **KP236765** |
| *T. americana* L. | A Sanchez 121 | Tolima, Colombia | WFU | **KP236795** | **KP236838** | **KP271198** | **KP271227** | **KP236769** |
| *T. americana* L. | A Sanchez 129 | Acre, Brazil | WFU | **KP236776** | **KP236817** | **KP271196** | **KP271231** | **KP236744** |
| *T. americana* L. | A Sanchez 139 | Acre, Brazil | WFU | **KP236778** | **KP236819** | **KP271199** | **KP271218** | NA |
| *T. americana* L. | A Sanchez 140 | Acre, Brazil | WFU | **KP236779** | **KP236820** | **KP271200** | **KP271215** | **KP236763** |
| *T. americana* L. | A Sanchez 176 | San Martin, Peru | WFU | **KP236782** | **KP236823** | **KP271213** | **KP271235** | **KP236762** |
| *T. cumingiana* Fisch. & C.A. Mey. ex C.A. Mey. | MA Luckow 4623 | Zulia, Venezuela | BH | **KP236790** | **KP236833** | **KP271211** | **KP271225** | **KP236764** |
| *T. cumingiana* Fisch. & C.A. Mey. ex C.A. Mey. | A Sanchez 122 | Cundinamarca, Colombia | WFU | **KP236796** | **KP236839** | **KP271208** | **KP271220** | **KP236745** |
| *T. dugandii* Brandbyge | A Sanchez 58 | Loreto, Peru | WFU | **KP236772** | **KP236813** | **KP271194** | NA | **KP236754** |
| *T. dugandii* Brandbyge | A Sanchez 203 | San Martin, Peru | WFU | **KP236784** | **KP236824** | **KP271212** | **KP271217** | NA |
| *T. gardneriana* Wedd. | MJN Rodal 426 | Pernambuco, Brazil | NY | NA | **KP236828** | **KP271192** | NA | NA |
| *T. longifolia* Huber | A Sanchez 188 | San Martin, Peru | WFU | **KP236783** | NA | **KP271207** | **KP271234** | NA |
| *T. melaenodendron* (Bertol.) Standl. & Steyerm. *subsp. colombiana* (Meisn.) Brandbyge | A Sanchez 110 | Antioquia, Colombia | WFU | **KP236793** | **KP236836** | **KP271201** | **KP271221** | NA |
| *T. melaenodendron* (Bertol.) Standl. & Steyerm. *subsp. colombiana* (Meisn.) Brandbyge | A Sanchez 119 | Valle del Cauca, Colombia | WFU | **KP236794** | **KP236837** | **KP271188** | **KP271216** | **KP236760** |
| *T. melaenodendron* (Bertol.) Standl. & Steyerm. | A Sanchez 405 | Guanacaste, Costa Rica | WFU | NA | **KP236829** | NA | **KP271228** | **KP236761** |
| *T. melaenodendron* (Bertol.) Standl. & Steyerm. | A Sanchez 407 | Punta Arenas, Costa Rica | WFU | **KP236789** | **KP236831** | **KP271214** | **KP271229** | **KP236755** |
| *T. melaenodendron* (Bertol.) Standl. & Steyerm. | A Sanchez 411 | Punta Arenas, Costa Rica | WFU | **KP236788** | **KP236830** | **KP271205** | **KP271224** | **KP236747** |
| *T. peruviana* Fisch. & Meyer ex C.A. Meyer | A Sanchez 171 | San Martin, Peru | WFU | **KP236780** | **KP236821** | **KP271190** | **KP271219** | NA |
| *T. peruviana* Fisch. & Meyer ex C.A. Meyer | A Sanchez 173 | San Martin, Peru | WFU | **KP236781** | **KP236822** | NA | **KP271223** | **KP236746** |
| *T. poeppigiana* Wedd. | A Sanchez 134 | Acre, Brazil | WFU | **KP236777** | **KP236818** | **KP271189** | NA | **KP236759** |
| *T. poeppigiana* Wedd. | A Sanchez 209 | Loreto, Peru | WFU | **KP236786** | **KP236826** | **KP271203** | **KP271236** | NA |
| *T. punctata* Standl. | A Sanchez 205 | San Martin, Peru | WFU | **KP236785** | **KP236825** | **KP271191** | **KP271226** | NA |
| *T. purdiei* Meisnn. in Mart. | A Sanchez 100 | Magdalena, Colombia | WFU | **KP236792** | **KP236835** | **KP271197** | NA | **KP236757** |
| *T. setosa* Rusby | AF Fuentes et al. 5351 | La Paz, Bolivia | MO | HQ693192 | **KP236840** | FJ154488 | HQ693135 | NA |
| *T. weigeltiana* (Rchb.) Kuntze | FA Michelangeli s.n. | Venezuela | WFU | HQ693193 | **KP236797** | HM137446 | **KP271232** | HQ693136 |
| *T. weigeltiana* (Rchb.) Kuntze | S Ramirez s.n. | Meta, Colombia | ANDES | **KP236775** | **KP236816** | **KP271209** | NA | **KP236766** |
| *T. weigeltiana* (Rchb.) Kuntze | A Sanchez 44 | Loreto, Peru | WFU | **KP236771** | **KP236812** | **KP271202** | NA | **KP236751** |
| *T. weigeltiana* (Rchb.) Kuntze | A Zambrano 8 | Leticia, Colombia | ANDES | **KP236773** | **KP236814** | **KP271206** | NA | **KP236752** |
| *T. weigeltiana* (Rchb.) Kuntze | A Zambrano 18 | Loreto, Peru | ANDES | **KP236774** | **KP236815** | **KP271193** | **KP271230** | **KP236768** |
