# Supplementary material for: Fidelity and Promiscuity in an Ant-Plant Mutualism: A Case Study of Triplaris and Pseudomyrmex
Source: PLoS One. 2015 Dec 2;10(12):e0143535. doi: 10.1371/journal.pone.0143535 (PMC4668088; doi:10.1371/journal.pone.0143535)
Supplement: S2 Table — Sequences obtained from Genbank are given with their respective site-specific numbers. New sequences generated for this study provide the following information: Taxon, collector(s) and collection number (#), location, and Genbank accession numbers. Specimens are part of Sanchez collection with duplicates in UCD (P. Ward collection). NA = not used in this study. In bold are the sequences generated in this study. For more details on collections by Sanchez see S3 Table. (DOCX) [file pone.0143535.s005.docx]

**S2** **Table**. Voucher information for ant DNA extractions used in this study. Sequences obtained from Genbank are given with their respective site-specific numbers. New sequences generated for this study provide the following information: Taxon, collector(s) and collection number (#), location, and Genbank accession numbers. Specimens are part of Sanchez collection with duplicates in UCD (P. Ward collection). NA= not used in this study. In bold are the sequences generated in this study. For more details on collections by A Sanchez see S3 Table.

| *Species* | *Collector, #* | *Location* | *COI* | *LR* |
| --- | --- | --- | --- | --- |
| *Pseudomyrmex* |  |  |  |  |
| *P. dendroicus* (Forel) | A Sanchez 31 | Madre de Dios, Peru | **KP271184** | **KP236801** |
| *P. dendroicus* (Forel) | A Sanchez 38 | Madre de Dios, Peru | **KP271186** | **KP236800** |
| *P. dendroicus* (Forel) | A Sanchez 40 | Madre de Dios, Peru | **KP271177** | NA |
| *P. elongatus* (Mayr) | A Sanchez 55 | Magdalena, Colombia | **KP271181** | **KP236804** |
| *P. gebellii* (Forel) | A Sanchez 68 | Valle del Cauca, Colombia | **KP271175** | **KP236806** |
| *P. longior* (Forel) | A Sanchez 67 | Antioquia, Colombia | **KP271187** | **KP236798** |
| *P. mordax* (Warming) | A Sanchez 51 | Santander, Colombia | **KP271176** | **KP236807** |
| *P. mordax* (Warming) | A Sanchez 58 | Bolivar, Colombia | **KP271185** | **KP236808** |
| *P. mordax* (Warming) | A Sanchez 70 | Tolima, Colombia | **KP271183** | **KP236802** |
| *P. triplaridis* (Forel) | A Sanchez 1 | Loreto, Peru | **KP271178** | **KP236803** |
| *P. triplaridis* (Forel) | A Sanchez 20 | Loreto, Peru | **KP271182** | **KP236799** |
| *P. triplarinus* (Weddell) | A Sanchez 9 | Loreto, Peru | **KP271179** | NA |
| *P. triplarinus* (Weddell) | A Sanchez 18 | Loreto, Peru | **KP271180** | NA |
| *P. viduus* (F. Smith) | A Sanchez 19 | Loreto, Peru | **KP271174** | **KP236805** |
|  |  |  |  |  |
| *Myrcidris epicharis* (Ward) | GenBank |  | NA | AY703785 |
| *P. ferrugineus* (F. Smith) | GenBank |  | FJ436818 | HM020792 |
| *P. flavicornis* (F. Smith) | GenBank |  | FJ436819 | AY703795 |
| *P. gracilis* (Fabricius) | GenBank |  | FJ436825 | AY703797 |
| *P. godmani* (Forel) | GenBank |  | FJ436820 | AY703796 |
| *P. haytianus* (Forel) | GenBank |  | FJ436826 | AY703798 |
| *P. major* (Forel) | GenBank |  | FJ436827 | FJ436878 |
| *P. mixtecus* (Ward) | GenBank |  | FJ436829 | HM020793 |
| *P. nigrocinctus* (Emery) | GenBank |  | FJ436830 | AY703802 |
| *P. nigropilosus* (Emery) | GenBank |  | FJ436833 | AY703803 |
| *P. peperi* (Forel) | GenBank |  | FJ436836 | HM020794 |
| *P. perboscii* (F. Smith) | GenBank |  | FJ436837 | FJ436886 |
| *P. satanicus* (Wheeler) | GenBank |  | FJ436840 | FJ436889 |
| *P. spinicola* (Emery) | GenBank |  | FJ436841 | FJ436890 |
| *P. tachigaliae* (Forel) | GenBank |  | NA | AY703814 |
| *P. viduus* (F. Smith) | GenBank |  | NA | AY703818 |
| *Tetraponera ambigua* (Emery) | GenBank |  | NA | AY703772 |
| *Tetraponera punctulata* F. Smith | GenBank |  | DQ373001 | AY703782 |
| *Tetraponera rufonigra* (Jerdon) | GenBank |  | FJ436846 | AY703783 |
