# Supplementary material for: Fidelity and Promiscuity in an Ant-Plant Mutualism: A Case Study of Triplaris and Pseudomyrmex
Source: PLoS One. 2015 Dec 2;10(12):e0143535. doi: 10.1371/journal.pone.0143535 (PMC4668088; doi:10.1371/journal.pone.0143535)
Supplement: S3 Table — # indicates the collection number for each organism. Ant species are color-coded. Plant species in bold indicate individuals associated with obligate ant mutualists. NI: indicates collections without information. (DOCX) [file pone.0143535.s006.docx]

**S3 Table**. Collections of *Triplaris* and associate ants. # indicates the collection number for each organism. Plant species in bold indicate individuals associated with obligate ant mutualists. *NI*: indicates collections without information.

| **Ant species** | **#** | **Plant species** | **#** | **Country** | **State** | **Lat.** | **Long.** | **Collector** |
| --- | --- | --- | --- | --- | --- | --- | --- | --- |
| **P. dendroicus** | PSW9076 | **T. americana** | *NI* | Bolivia | Beni | -14.8 | -66.38 | PS Ward |
| **P. dendroicus** | *NI* | **T. americana** | *NI* | Bolivia | Santa Cruz | -14.73 | -62.8 | P Bettella |
| **P. dendroicus** | S84 | **T. americana** | S136 | Brazil | Acre | -10.08 | -67.55 | A Sanchez |
| **P. dendroicus** | S99 | **T. americana** | S155 | Brazil | Acre | -9.95 | -67.81 | A Sanchez |
| **P. dendroicus** | S100 | **T. americana** | S156 | Brazil | Acre | -9.93 | -67.89 | A Sanchez |
| **P. dendroicus** | S86 | **T. americana** | S138 | Brazil | Acre | -10.02 | -67.56 | A Sanchez |
| **P. dendroicus** | S87 | **T. americana** | S139 | Brazil | Acre | -9.95 | -67.86 | A Sanchez |
| **P. dendroicus** | S89 | **T. americana** | S141 | Brazil | Acre | -9.75 | -67.67 | A Sanchez |
| **P. dendroicus** | S90 | **T. americana** | S145 | Brazil | Acre | -9.75 | -67.67 | A Sanchez |
| **P. dendroicus** | S91 | **T. americana** | S146 | Brazil | Acre | -9.71 | -68.09 | A Sanchez |
| **P. dendroicus** | S92 | **T. americana** | S147 | Brazil | Acre | -9.71 | -68.09 | A Sanchez |
| **P. dendroicus** | S93 | **T. americana** | S148 | Brazil | Acre | -9.71 | -68.11 | A Sanchez |
| **P. dendroicus** | S94 | **T. americana** | S149 | Brazil | Acre | -9.61 | -68.25 | A Sanchez |
| **P. dendroicus** | S95 | **T. americana** | S150 | Brazil | Acre | -9.57 | -68.28 | A Sanchez |
| **P. dendroicus** | S96 | **T. americana** | S151 | Brazil | Acre | -9.49 | -68.35 | A Sanchez |
| **P. dendroicus** | S97 | **T. americana** | S153 | Brazil | Acre | -9.46 | -68.38 | A Sanchez |
| **P. dendroicus** | *NI* | **T. americana** | *NI* | Colombia | Villavicencio | 4.28 | -74.05 | Schremmer 1984; Ward 1999 |
| **P. dendroicus** | PSW11323 | **T. americana** | *NI* | Ecuador | Napo | -1.07 | -77.62 | PS Ward |
| **P. dendroicus** | *NI* | **T. americana** | B33476 | Ecuador | Sucumbios | -0.25 | -76.35 | J Brandbyge |
| **P. dendroicus** | *NI* | **T. americana** | *NI* | Peru | Huanuco | -9.58 | -74.8 | NL Toft |
| **P. dendroicus** | S136 | **T. americana** | S210 | Peru | Loreto | -5.12 | -75.73 | A Sanchez |
| **P. dendroicus** | S29 | **T. americana** | S69 | Peru | Madre de Dios | -12.84 | -69.29 | A Sanchez |
| **P. dendroicus** | S31 | **T. americana** | S74 | Peru | Madre de Dios | -12.84 | -69.29 | A Sanchez |
| **P. dendroicus** | S32 | **T. americana** | S75 | Peru | Madre de Dios | -12.84 | -69.29 | A Sanchez |
| **P. dendroicus** | S33 | **T. americana** | S76 | Peru | Madre de Dios | -12.84 | -69.29 | A Sanchez |
| **P. dendroicus** | S34 | **T. americana** | S77 | Peru | Madre de Dios | -12.84 | -69.28 | A Sanchez |
| **P. dendroicus** | S35 | **T. americana** | S78 | Peru | Madre de Dios | -12.84 | -69.29 | A Sanchez |
| **P. dendroicus** | S36 | **T. americana** | S79 | Peru | Madre de Dios | -12.84 | -69.29 | A Sanchez |
| **P. dendroicus** | S37 | **T. americana** | S80 | Peru | Madre de Dios | -12.57 | -70.09 | A Sanchez |
| **P. dendroicus** | S38 | **T. americana** | S82 | Peru | Madre de Dios | -12.57 | -70.09 | A Sanchez |
| **P. dendroicus** | S40 | **T. americana** | S84 | Peru | Madre de Dios | -12.57 | -70.1 | A Sanchez |
| **P. dendroicus** | S41 | **T. americana** | S85 | Peru | Madre de Dios | -12.56 | -70.09 | A Sanchez |
| **P. dendroicus** | S42 | **T. americana** | S86 | Peru | Madre de Dios | -12.56 | -70.09 | A Sanchez |
| **P. dendroicus** | S46A | **T. americana** | S91A | Peru | Madre de Dios | -12.57 | -70.09 | A Sanchez |
| **P. dendroicus** | *NI* | **T. americana** | *NI* | Peru | Madre de Dios | -11.85 | -71.32 | DW Davidson |
| **P. dendroicus** | S126 | **T. americana** | S198 | Peru | San Martín | -5.98 | -77.22 | A Sanchez |
| **P. dendroicus** | *NI* | **T. americana** | *NI* | Peru | Tarapoto | *NI* | *NI* | Ule 1906; Ward 1999 |
| **P. mordax** | S58 | **T. americana** | S103 | Colombia | Bolivar | 10.14 | -75.04 | A Sanchez |
| **P. mordax** | S52 | **T. americana** | S97 | Colombia | Cesar | 8.62 | -73.68 | A Sanchez |
| **P. mordax** | S53 | **T. americana** | S98 | Colombia | Cesar | 9.14 | -73.66 | A Sanchez |
| **P. mordax** | S62 | **T. americana** | S107 | Colombia | Cordoba | 9.3 | -75.88 | A Sanchez |
| **P. mordax** | S64 | **T. americana** | S109 | Colombia | Cordoba | 8.59 | -75.73 | A Sanchez |
| **P. mordax** | S50 | **T. americana** | S95 | Colombia | Santander | 6.86 | -73.75 | A Sanchez |
| **P. mordax** | S51 | **T. americana** | S96 | Colombia | Santander | 7.6 | -73.55 | A Sanchez |
| **P. mordax** | *NI* | **T. americana** | *NI* | Colombia | Since | 8.75 | -66.27 | Schremmer 1984; Ward 1999 |
| **P. mordax** | S59 | **T. americana** | S104 | Colombia | Sucre | 9.58 | -75.19 | A Sanchez |
| **P. mordax** | S61 | **T. americana** | S106 | Colombia | Sucre | 9.41 | -75.09 | A Sanchez |
| **P. mordax** | S70 | **T. americana** | S121 | Colombia | Tolima | 4.07 | -74.94 | A Sanchez |
| **P. mordax** | *NI* | **T. americana** | *NI* | Venezuela | Miranda | 10.27 | -66.33 | Jaffé et al. 1986 |
| **P. mordax** | *NI* | **T. cumingiana** | *NI* | Panama | Gatun Lake | 8.95 | -79.8 | Wheeler 1942 |
| **P. mordax** | S54 | **T. purdiei** | S99 | Colombia | Cesar | 9.23 | -73.52 | A Sanchez |
| **P. triplaridis** | S135 | **T. americana** | S208 | Peru | Loreto | -5.12 | -75.73 | A Sanchez |
| **P. triplaridis** | S115 | **T. americana** | S184 | Peru | San Martín | -7.35 | -76.68 | A Sanchez |
| **P. triplaridis** | *NI* | **T. dugandii** | B36208 | Ecuador | Sucumbios | -0.25 | -76.35 | J Brandbyge |
| **P. triplaridis** | S112 | **T. dugandii** | S181 | Peru | San Martín | -6.72 | -76.25 | A Sanchez |
| **P. triplaridis** | S121 | **T. dugandii** | S190 | Peru | San Martín | -6.57 | -76.13 | A Sanchez |
| **P. triplaridis** | S128 | **T. dugandii** | S200 | Peru | San Martín | -6.06 | -77.26 | A Sanchez |
| **P. triplaridis** | *NI* | **T. dugandii** | S201 | Peru | San Martín | -6.05 | -77.26 | A Sanchez |
| **P. triplaridis** | S130 | **T. dugandii** | S203 | Peru | San Martín | -6.22 | -76.82 | A Sanchez |
| **P. triplaridis** | S102 | **T. peruviana** | S171 | Peru | San Martín | -6.55 | -76.34 | A Sanchez |
| **P. triplaridis** | S103 | **T. peruviana** | S172 | Peru | San Martín | -6.55 | -76.34 | A Sanchez |
| **P. triplaridis** | S104 | **T. peruviana** | S173 | Peru | San Martín | -6.59 | -76.31 | A Sanchez |
| **P. triplaridis** | S114 | **T. peruviana** | S183 | Peru | San Martín | -6.92 | -76.38 | A Sanchez |
| **P. triplaridis** | S116 | **T. peruviana** | S185 | Peru | San Martín | -7.12 | -76.69 | A Sanchez |
| **P. triplaridis** | S131 | **T. setosa** | S204 | Peru | San Martín | -6.41 | -76.62 | A Sanchez |
| **P. triplaridis** | S83 | **T. weigeltiana** | S135 | Brazil | Acre | -10.08 | -67.54 | A Sanchez |
| **P. triplaridis** | S85 | **T. weigeltiana** | S137 | Brazil | Acre | -10.09 | -67.55 | A Sanchez |
| **P. triplaridis** | *NI* | **T. weigeltiana** | *NI* | Ecuador | Sucumbios | -0.27 | -76.33 | J Brandbyge |
| **P. triplaridis** | *NI* | **T. weigeltiana** | B36192 | Ecuador | Sucumbios | -0.25 | -76.35 | J Brandbyge |
| **P. triplaridis** | *NI* | **T. weigeltiana** | *NI* | Guyana | Camaria | 6.65 | -59.57 | Wheeler 1942 |
| **P. triplaridis** | *NI* | **T. weigeltiana** | *NI* | Guyana | Mahaica | 6.25 | -57.55 | HE Box |
| **P. triplaridis** | *NI* | **T. weigeltiana** | *NI* | Peru | Iquitos | *NI* | *NI* | Ule 1906; Ward 1999 |
| **P. triplaridis** | S01 | **T. weigeltiana** | S40 | Peru | Loreto | -3.74 | -73.25 | A Sanchez |
| **P. triplaridis** | S03 | **T. weigeltiana** | S41 | Peru | Loreto | -3.83 | -73.38 | A Sanchez |
| **P. triplaridis** | S04 | **T. weigeltiana** | S43 | Peru | Loreto | -3.85 | -73.43 | A Sanchez |
| **P. triplaridis** | S06 | **T. weigeltiana** | S45 | Peru | Loreto | -3.84 | -73.4 | A Sanchez |
| **P. triplaridis** | S07b | **T. weigeltiana** | S47 | Peru | Loreto | -3.63 | -73.34 | A Sanchez |
| **P. triplaridis** | S08 | **T. weigeltiana** | S48 | Peru | Loreto | -3.63 | -73.34 | A Sanchez |
| **P. triplaridis** | S11 | **T. weigeltiana** | S51 | Peru | Loreto | -3.92 | -73.32 | A Sanchez |
| **P. triplaridis** | S16 | **T. weigeltiana** | S56 | Peru | Loreto | -3.59 | -73.12 | A Sanchez |
| **P. triplaridis** | S20 | **T. weigeltiana** | S60 | Peru | Loreto | -3.49 | -73.09 | A Sanchez |
| **P. triplaridis** | S21 | **T. weigeltiana** | S61 | Peru | Loreto | -3.48 | -73.08 | A Sanchez |
| **P. triplaridis** | S22 | **T. weigeltiana** | S62 | Peru | Loreto | -3.43 | -73.04 | A Sanchez |
| **P. triplaridis** | S24 | **T. weigeltiana** | S64 | Peru | Loreto | -3.4 | -73 | A Sanchez |
| **P. triplaridis** | S28 | **T. weigeltiana** | S68 | Peru | Loreto | -4.46 | -73.44 | A Sanchez |
| **P. triplaridis** | S133 | **T. weigeltiana** | S206 | Peru | Loreto | -5.12 | -75.69 | A Sanchez |
| **P. triplaridis** | S124 | **T. weigeltiana** | S194 | Peru | San Martín | -6.04 | -77.12 | A Sanchez |
| **P. triplaridis** | *NI* | **T. weigeltiana** | S196 | Peru | San Martín | -5.98 | -77.21 | A Sanchez |
| **P. triplaridis** | S108 | **T. weigeltiana** | S177 | Peru | San Martín | -6.71 | -76.21 | A Sanchez |
| **P. triplarinus** | PSW9075 | **T. americana** | *NI* | Bolivia | Beni | -14.8 | -66.38 | PS Ward |
| **P. triplarinus** | *NI* | **T. americana** | *NI* | Bolivia | Santa Cruz | -17.85 | -63.17 | P Bettella |
| **P. triplarinus** | S72 | **T. americana** | S123 | Brazil | Acre | -9.98 | -66.8 | A Sanchez |
| **P. triplarinus** | S74 | **T. americana** | S125 | Brazil | Acre | -9.98 | -66.77 | A Sanchez |
| **P. triplarinus** | S75 | **T. americana** | S126 | Brazil | Acre | -9.98 | -66.77 | A Sanchez |
| **P. triplarinus** | S76 | **T. americana** | S127 | Brazil | Acre | -10.01 | -66.77 | A Sanchez |
| **P. triplarinus** | S77 | **T. americana** | S128 | Brazil | Acre | -10.02 | -66.77 | A Sanchez |
| **P. triplarinus** | S88 | **T. americana** | S140 | Brazil | Acre | -9.75 | -67.67 | A Sanchez |
| **P. triplarinus** | S78 | **T. americana** | S129 | Brazil | Acre | -10.02 | -66.78 | A Sanchez |
| **P. triplarinus** | S79 | **T. americana** | S130 | Brazil | Acre | -10.05 | -66.81 | A Sanchez |
| **P. triplarinus** | S80 | **T. americana** | S131 | Brazil | Acre | -10.08 | -66.84 | A Sanchez |
| **P. triplarinus** | S81 | **T. americana** | S132 | Brazil | Acre | -10.08 | -66.86 | A Sanchez |
| **P. triplarinus** | S82 | **T. americana** | S133 | Brazil | Acre | -10.01 | -66.78 | A Sanchez |
| **P. triplarinus** | S89B | **T. americana** | S143 | Brazil | Acre | -9.76 | -67.67 | A Sanchez |
| **P. triplarinus** | *NI* | **T. americana** | *NI* | Brazil | Mato Grosso | -17.27 | -56.98 | Oliveira et al. 1987 |
| **P. triplarinus** | *NI* | **T. americana** | *NI* | Brazil | Rondonia | -8.75 | -63.47 | Unknown |
| **P. triplarinus** | *NI* | **T. americana** | *NI* | Peru | Huanuco | -9.58 | -74.8 | NL Toft |
| **P. triplarinus** | S106 | **T. americana** | S175 | Peru | San Martín | -6.72 | -76.21 | A Sanchez |
| **P. triplarinus** | S107 | **T. americana** | S176 | Peru | San Martín | -6.72 | -76.21 | A Sanchez |
| **P. triplarinus** | S117 | **T. americana** | S186 | Peru | San Martín | -6.37 | -76.54 | A Sanchez |
| **P. triplarinus** | S73 | **T. dugandii** | S124 | Brazil | Acre | -9.98 | -66.8 | A Sanchez |
| **P. triplarinus** | S09 | **T. dugandii** | S49 | Peru | Loreto | -3.63 | -73.34 | A Sanchez |
| **P. triplarinus** | S18 | **T. dugandii** | S58 | Peru | Loreto | -3.54 | -73.08 | A Sanchez |
| **P. triplarinus** | S26 | **T. dugandii** | S66 | Peru | Loreto | -4.48 | -73.51 | A Sanchez |
| **P. triplarinus** | S119 | **T. cf. longifolia** | S188 | Peru | San Martín | -6.46 | -76.35 | A Sanchez |
| **P. triplarinus** | S122 | **T. cf. longifolia** | S191 | Peru | San Martín | -6.6 | -76.15 | A Sanchez |
| **P. triplarinus** | *NI* | **T. cf. longifolia** | S193 | Peru | San Martín | -6.61 | -76.16 | A Sanchez |
| **P. triplarinus** | S120 | **T. longifolia** | S189 | Peru | San Martín | -6.62 | -76.17 | A Sanchez |
| **P. triplarinus** | S123 | **T. longifolia** | S192 | Peru | San Martín | -6.6 | -76.15 | A Sanchez |
| **P. triplarinus** | S105 | **T. peruviana** | S174 | Peru | San Martín | -6.59 | -76.31 | A Sanchez |
| **P. triplarinus** | S111 | **T. peruviana** | S180 | Peru | San Martín | -6.73 | -76.25 | A Sanchez |
| **P. triplarinus** | S118 | **T. peruviana** | S187 | Peru | San Martín | -6.44 | -76.5 | A Sanchez |
| **P. triplarinus** | S132 | **T. punctata** | S205 | Peru | San Martín | -6.47 | -76.32 | A Sanchez |
| **P. triplarinus** | S98 | **T. weigeltiana** | S154 | Brazil | Acre | -9.23 | -68.53 | A Sanchez |
| *P. ultrix** | PSW11393 | **T. dugandii** | *NI* | Ecuador | Napo | -0.8 | -77.78 | PS Ward |
| **P. viduus** | *NI* | T. americana | L4635 | Venezuela | Caracas | 10.48 | -66.9 | MA Luckow |
| **P. viduus** | S140 | T. melaenodendron | S402 | Costa Rica | Guanacaste | 10.34 | -85.27 | A Sanchez |
| **P. viduus** | S141 | T. melaenodendron | S403 | Costa Rica | Guanacaste | 10.17 | -85.59 | A Sanchez |
| **P. viduus** | S142 | T. melaenodendron | S405 | Costa Rica | Guanacaste | 10.8 | -85.65 | A Sanchez |
| **P. viduus** | S143 | T. melaenodendron | S407 | Costa Rica | Guanacaste | 10.97 | -85.69 | A Sanchez |
| **P. viduus** | *NI* | T. melaenodendron | *NI* | Costa Rica | Guanacaste | 10.33 | -85.2 | DH Janzen |
| **P. viduus** | *NI* | T. melaenodendron | *NI* | Costa Rica | Guanacaste | 10.35 | -85.35 | J Longino |
| **P. viduus** | *NI* | T. weigeltiana | *NI* | Guyana | Cuyuni | 6.8 | -59.75 | L Gillespie |
| **P. viduus** | *NI* | T. weigeltiana | *NI* | Guyana | Mahaica | 6.25 | -57.55 | HE Box |
| **P. viduus** | S19 | T. weigeltiana | S59 | Peru | Loreto | -3.54 | -73.08 | A Sanchez |
| **Other Pseudomyrmex** | |  |  |  |  |  |  |  |
| **P. elongatus** | S55 | T. purdiei | S100 | Colombia | Magdalena | 9.57 | -73.90 | A Sanchez |
| **P. elongatus** | S60 | T. americana | S104 | Colombia | Sucre | 9.41 | -75.23 | A Sanchez |
| **P. gebelli** | S68 | T. melaenodendron | S119 | Colombia | Valle del Cauca | 4.07 | -77.23 | A Sanchez |
| **P. longior** | S67 | T. melaenodendron | S118 | Colombia | Antioquia | 4.01 | -75.77 | A Sanchez |
| **Other genera** | |  |  |  |  |  |  |  |
| **Azteca sp.** | S49 | T. americana | S94 | Colombia | Santander | 6.86 | -73.75 | A Sanchez |
| **Azteca sp.** | S110 | T. peruviana | S179 | Peru | San Martin | -5.27 | -76.25 | A Sanchez |
| **Azteca sp.** | S45 | T. poeppigiana | S90a | Peru | Madre de Dios | -11.43 | -70.10 | A Sanchez |
| **Azteca sp.** | *NI* | T. poeppigiana | S209 | Peru | Loreto | -4.88 | -75.73 | A Sanchez |
| **Azteca sp.** | *NI* | T. poeppigiana | S71 | Peru | Madre de Dios | -11.17 | -69.29 | A Sanchez |
| **Azteca sp.** | *NI* | T. poeppigiana | S72 | Peru | Madre de Dios | -11.16 | -69.29 | A Sanchez |
| **Azteca sp.** | S30 | T. poeppigiana | S73 | Peru | Madre de Dios | -11.16 | -69.29 | A Sanchez |
| **Azteca sp.** | S44 | T. poeppigiana | S89 | Peru | Madre de Dios | -11.43 | -70.09 | A Sanchez |
| **Azteca sp.** | *NI* | T. poeppigiana | S88 | Peru | Madre de Dios | -11.43 | -70.09 | A Sanchez |
| **Azteca sp.** | S39 | T. poeppigiana | S83 | Peru | Madre de Dios | -11.42 | -70.09 | A Sanchez |
| **Azteca sp.** | S113 | T. poeppigiana | S182 | Peru | San Martin | -5.07 | -76.36 | A Sanchez |
| **Azteca sp.** | S15 | T. weigeltiana | S55 | Peru | Loreto | -2.16 | -73.45 | A Sanchez |
| **Azteca sp.** | S25 | T. weigeltiana | S65 | Peru | Loreto | -2.08 | -73.32 | A Sanchez |
| **Azteca sp.** | S23 | T. weigeltiana | S63 | Peru | Loreto | -2.10 | -73.32 | A Sanchez |
| **Azteca sp.** | S5 | T. weigeltiana | S44 | Peru | Loreto | -2.41 | -73.26 | A Sanchez |
| **Azteca sp.** | S12 | T. weigeltiana | S52 | Peru | Loreto | -2.60 | -73.02 | A Sanchez |
| **Azteca sp.** | S10 | T. weigeltiana | S50 | Peru | Loreto | -3.52 | -73.02 | A Sanchez |
| **Azteca sp.** | S125 | T. weigeltiana | S197 | Peru | San Martin | -4.02 | -77.22 | A Sanchez |
| **Azteca sp.** | S129 | T. weigeltiana | S202 | Peru | San Martin | -5.78 | -77.16 | A Sanchez |
| **Camponotus sexguttatus** | S17 | T. weigeltiana | S53 | Peru | Loreto | -2.46 | -73.11 | A Sanchez |
| **Cephalotes ramiphilus** | S13 | T. weigeltiana | S57 | Peru | Loreto | -2.11 | -73.32 | A Sanchez |
| **Crematogaster sp.** | S69 | T. americana | S120 | Colombia | Tolima | 4.07 | -74.94 | A Sanchez |
| **Crematogaster sp.** | S71 | T. cumingiana | S122 | Colombia | Cundinamarca | 4.49 | -74.61 | A Sanchez |
| **Crematogaster sp.** | S66 | T. melaenodendron | S116 | Colombia | Antioquia | 6.59 | -75.85 | A Sanchez |
| **Crematogaster sp.** | S65 | T. melaenodendron | S117 | Colombia | Antioquia | 6.54 | -75.85 | A Sanchez |
| **Crematogaster sp.** | S145 | T. melaenodendron | S410 | Costa Rica | Puntarenas | 8.53 | -84.61 | A Sanchez |
| **Crematogaster sp.** | S57 | T. purdiei | S102 | Colombia | Atlántico | 10.71 | -73.89 | A Sanchez |
| **Crematogaster sp.** | S56 | T. purdiei | S101 | Colombia | Magdalena | 10.14 | -74.75 | A Sanchez |
| **Crematogaster sp.** | S2 | T. weigeltiana | S41 | Peru | Loreto | -2.17 | -73.38 | A Sanchez |
| **Crematogaster sp.** | S14 | T. weigeltiana | S54 | Peru | Loreto | -2.21 | -73.32 | A Sanchez |
| **Crematogaster sp.** | S27 | T. weigeltiana | S67 | Peru | Loreto | -3.54 | -73.49 | A Sanchez |
| **Dolychoderus bidens** | S07 | T. weigeltiana | S46 | Peru | Loreto | -2.37 | -73.39 | A Sanchez |
| **NO ANTS** |  |  |  |  |  |  |  | A Sanchez |
|  |  | T. cumingiana | S195 | Peru | San Martin | -6.04 | -77.12 | A Sanchez |
|  |  | T. cumingiana | S90 | Colombia | Cundinamarca | 5.17 | -74.66 | A Sanchez |
|  |  | T. cumingiana | S92 | Colombia | Cundinamarca | 5.19 | -74.7 | A Sanchez |
|  |  | T. dugandii | S142 | Brazil | Acre | -9.75 | -67.67 | A Sanchez |
|  |  | T. dugandii | S144 | Brazil | Acre | -9.76 | 67.67 | A Sanchez |
|  |  | T.melaenodendron | S110 | Colombia | Antioquia | 6.27 | -75.57 | A Sanchez |
|  |  | T.melaenodendron | S111 | Colombia | Antioquia | 6.27 | -75.57 | A Sanchez |
|  |  | T.melaenodendron | S112 | Colombia | Antioquia | 6.49 | -75.79 | A Sanchez |
|  |  | T.melaenodendron | S113 | Colombia | Antioquia | 6.49 | -75.79 | A Sanchez |
|  |  | T.melaenodendron | S114 | Colombia | Antioquia | 6.56 | -75.84 | A Sanchez |
|  |  | T.melaenodendron | S115 | Colombia | Antioquia | 6.56 | -75.84 | A Sanchez |
|  |  | T.melaenodendron | S409 | Costa Rica | Puntarenas | 8.53 | -83.39 | A Sanchez |
|  |  | T.melaenodendron | S411 | Costa Rica | Puntarenas | 8.99 | -83.60 | A Sanchez |
|  |  | T. poeppigiana | S134 | Brazil | Acre | -10.01 | -66.78 | A Sanchez |
|  |  | T. weigeltiana | S42 | Peru | Loreto | -3.85 | -73.42 | A Sanchez |

* species not sequenced for the phylogeny

**References**

Jaffé K, Lopez ME, Aragort W. On the communication systems of the ants *Pseudomyrmex termitarius* and *P. triplarinus*. Insec Soc 1986; 33: 105–117.

Oliveira PS, Oliveira Filho AT, Cintra R. Ant Foraging On Ant–Inhabited *Triplaris* (Polygonaceae) in Western Brazil a Field Experiment Using Live Termite Baits. J Trop Ecol. 1987; 3: 193–200.

Schremmer F. Untersuchungen und Beobachtungen zur Ökoethologie der Pflanzenameise *Pseudomyrmex triplarinus*, welche die Ameisenbäume der Gattung *Triplaris* bewohnt. Zool Jahr Abt Syst Oekol Geogr Tiere. 1984; 111: 385–410.

Ule E. Ameisenpflanzen. Bot Jahrb Syst. 1906; 37: 335–352.

Ward PS. Systematics, biogeography and host plant associations of the *Pseudomyrmex viduus* group (Hymenoptera: Formicidae), *Triplaris*- and *Tachigali*-inhabiting ants. Zool J Linn Soc. 1999; 126: 451–540.

Wheeler WM. Studies of Neotropical Ant-Plants and their Ants. Bull Mus Comp Zool 1942; 90: 41–65.
